# Synthesis and Antibacterial Activity of Mono- and Bi-Cationic Pyridinium 1,2,4-Oxadiazoles and Triazoles

**DOI:** 10.3390/ijms25010377

**Published:** 2023-12-27

**Authors:** Sara Amata, Cinzia Calà, Carla Rizzo, Ivana Pibiri, Mariangela Pizzo, Silvestre Buscemi, Antonio Palumbo Piccionello

**Affiliations:** 1 Department of Biological, Chemical and Pharmaceutical Sciences and Technologies (STEBICEF), University of Palermo, Viale delle Scienze, Ed. 17, 90128 Palermo, Italy; sara.amata01@unipa.it (S.A.); carla.rizzo03@unipa.it (C.R.); ivana.pibiri@unipa.it (I.P.); silvestre.buscemi@unipa.it (S.B.); 2 Department of Health Promotion, Mother and Child Care, Internal Medicine and Medical Specialties “G D’Alessandro”, University of Palermo, Via Del Vespro 133, 90127 Palermo, Italy; cinzia.cala@unipa.it (C.C.); mariangela.pizzo22@gmail.com (M.P.); 3 Microbiology and Virology Unit, AOU Policlinico “P. Giaccone”, 90127 Palermo, Italy

**Keywords:** antibiotics resistance, pyridinium salts, oxadiazole

## Abstract

One of the main causes of mortality in humans continues to be infectious diseases. Scientists are searching for new alternatives due to the fast increase in resistance of some harmful bacteria to the frontline antibiotics. To effectively treat pathogenic infections, it is crucial to design antibiotics that can prevent the development of pathogenic resistance. For this purpose, a set of 39 quaternary pyridinium and bis-pyridinium salts with different lengths of side alkyl or fluorinated chains, heterocyclic spacers, and counter ions were tested on diverse reference bacterial ATCC (American Type Culture Collection) strains, such as *S. aureus* and *E. coli*. Subsequently, 6 out of the 39 pyridinium salts showing relevant MIC (Minimum Inhibitory Concentration) values were tested on clinically isolated, resistant strains of *S. aureus*, *S. epidermids*, *S. haemolyticus*, *K. pneumoniae*, *A. baumannii*, and *P. aeruginosa*. Additional tests have been performed to assess if the minimum concentration detected through MIC assay may limit the growth of biofilms.

## 1. Introduction

The increasing number of bacteria that are resistant to many classes of frequently administered antibiotics creates an important threat to the therapeutic use of antimicrobial substances and, consequently, the successful treatment of infections caused by bacteria [1,2,3,4]. Unchecked antimicrobial resistance will have a devastating financial impact on food systems, livelihoods, and health care costs [5,6]. *Enterococcus* spp., *S. aureus*, *K. pneumoniae*, *A. baumannii*, *P. aeruginosa*, *Enterobacter* spp., *Acinetobacter* spp., *Salmonella* spp., and *Streptococcus pneumoniae* are the resistant microorganisms that are becoming more and more involved in the majority of bacterial infections [7].

It is crucial to significantly improve public awareness about microorganisms and antibiotic resistance [8]. Eventually, it is necessary to maintain a steady stream of new structural classes of antibiotics that are unaffected by established or recognized mechanisms of resistance [9,10].

Cationic antimicrobials have been employed for more than a century in both the prevention of infections and several consumer items, and it is frequently believed that they have a single, universal mechanism of action that is directed at biological membranes. One of the most popular antiseptics found in dental care products is cethylpyridinium chloride (CPC), for which the antibacterial activity was first described in 1939 by the laboratories of the Wm. S. Merrell Company in Cincinnati, Ohio [11,12]. Its structure consists of a quaternary nitrogen bearing one hydrophobic side chain [13]. The alkyl chain regulates the antibacterial activity of the CPC by changing its hydrophobicity. In fact, the C12-16 CPC salts showed the best bactericidal activity. CPC impacts the cell by interfering with its osmoregulation and homeostasis at low doses. When CPC is present in high amounts, the membranes fall apart, thus allowing the cytoplasmic contents to flow out.

Gram-negative and Gram-positive microorganisms are both susceptible to the antimicrobial effects of quaternary pyridinium salts with long alkyl chains [14]. As sanitizing and antiseptic agents, ingredients in cosmetic formulations, and germicides and fungicides today, cationic compounds with antibacterial capabilities continue to play a significant role [15]. In addition to the pyridine, the inclusion of another heterocycle to the bioactive molecules was proved to be advantageous as it enhanced their antibacterial and antiviral characteristics and broadened their range of action [16]. A hydrophobic group that is directly or indirectly linked to a positively charged nitrogen atom is what distinguishes cationic surfactants from other classes of compounds. In fact, the length of the alkyl side chain affects the antimicrobial activity of these quaternary compounds. The maximum activity against Gram-negative strains is reached for an alkyl chain of 12 to 14 carbon atoms, while the maximum activity against Gram-positive strains is reached for an alkyl chain of 14 to 16 [13,17,18]. In addition, they are more soluble in water thanks to the hydrophilic component, which is typically the anion counterpart, such as a chloride (Cl) or bromide (Br). The mechanism has little impact on the resistance or sensitivity of bacteria because the hydrophobic long alkyl chain component can enter inside the nonpolar cell membrane. This affects the permeability of the cell membrane and kills the bacteria cells [19]. Recently, a different class of amphiphilic molecules gained attention in the pharmaceutical industry. These classes of new compounds are the so-called bipolar or gemini surfactants [20]. In particular, they show two hydrophilic head groups connected through a spacer, which could be, for example, a long alkyl chain or a heterocycle. In turn, each head group could have various alkyl chain lengths bonded to the nitrogen atom. The dimeric surfactants in general show better active properties than their mono-cationic conventional analogues [21]. For a very long time, many ionic liquids (ILs), including CPC, have been widely utilized as antiseptics [22]. At first, ILs were viewed as eco-friendly solvents that could take the place of conventional, hazardous organic solvents in a variety of chemical processes. But, as proof of the strong biological activity of different classes of ILs began to emerge, these compounds started to be considered as potential novel medications and drug-like molecules. In particular, the antibacterial activity of ILs has received much interest, and their potential uses in medicine as well as in the environment have been suggested [23,24,25]. In this work, the antibacterial activity of different mono- and bi-pyridinium salts with different lengths of the alkyl chain, fluorinated or not, and with different counter ions, was evaluated. Some of the *N*-alkylpyridinium salts and *N*-perfluoroalkylpiridinium salts taken into consideration have been previously reported for different applications, such as ILs or ionic liquid crystals (ILCs) [26,27,28,29,30,31,32]. These organic salts have an azole ring as a spacer (specifically, 1,2,4-oxadiazole or 1,2,4-triazole). It may be possible to modify the physical and chemical features of these compounds by carefully adjusting various factors, such as the heterocycle, the length of the chain, and its position inside the heterocyclic ring.

## 2. Results and Discussion

### 2.1. Synthesis

**Scheme 1:**
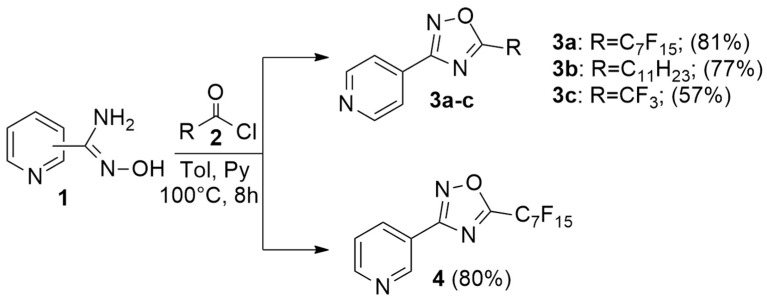
Synthesis of compounds **3** and **4**.

To obtain the 5-alkyl- or perfluoroalkyl-3-[4′-pyridyl]-1,2,4-oxadiazoles **3a**–**c** and 5-perfluoroheptyl-3-[3′-pyridyl]-1,2,4-oxadiazole **4**, a classic method of synthesis of 1,2,4-oxadiazole was performed [33]. This was achieved through the reaction of the amidoxime **1** (nicotinoyl or isonicotinoyl amidoxime) with the appropriate acyl or perfluoroacyl chloride (Scheme 1).

**Scheme 2:**
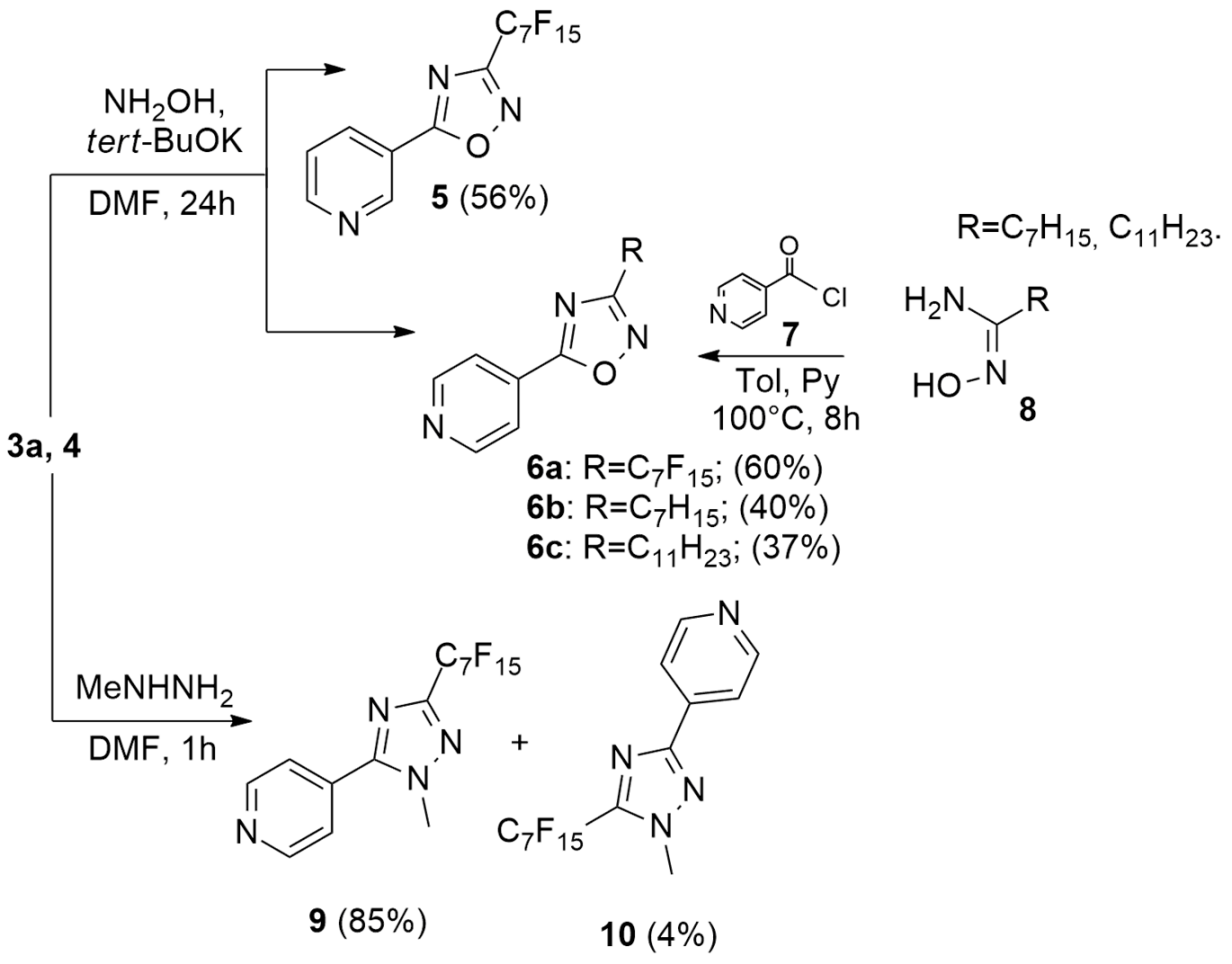
Synthesis of compounds **5**, **6**, **9**, and **10**.

The 5-perfluoroheptyloxadiazole derivates **3a** and **4** gave, through an ANRORC (Addition of a Nucleophile with Ring Opening and Ring Closure) [32,33,34] rearrangement, their 3-regioisomer **5** and **6a** 3-perfluoroheptyl-5-[3′-pyridyl]-1,2,4-oxadiazole and 3-perfluoroheptyl-5-[4′-pyridyl]-1,2,4-oxadiazole, respectively (Scheme 2).

**Scheme 3:**
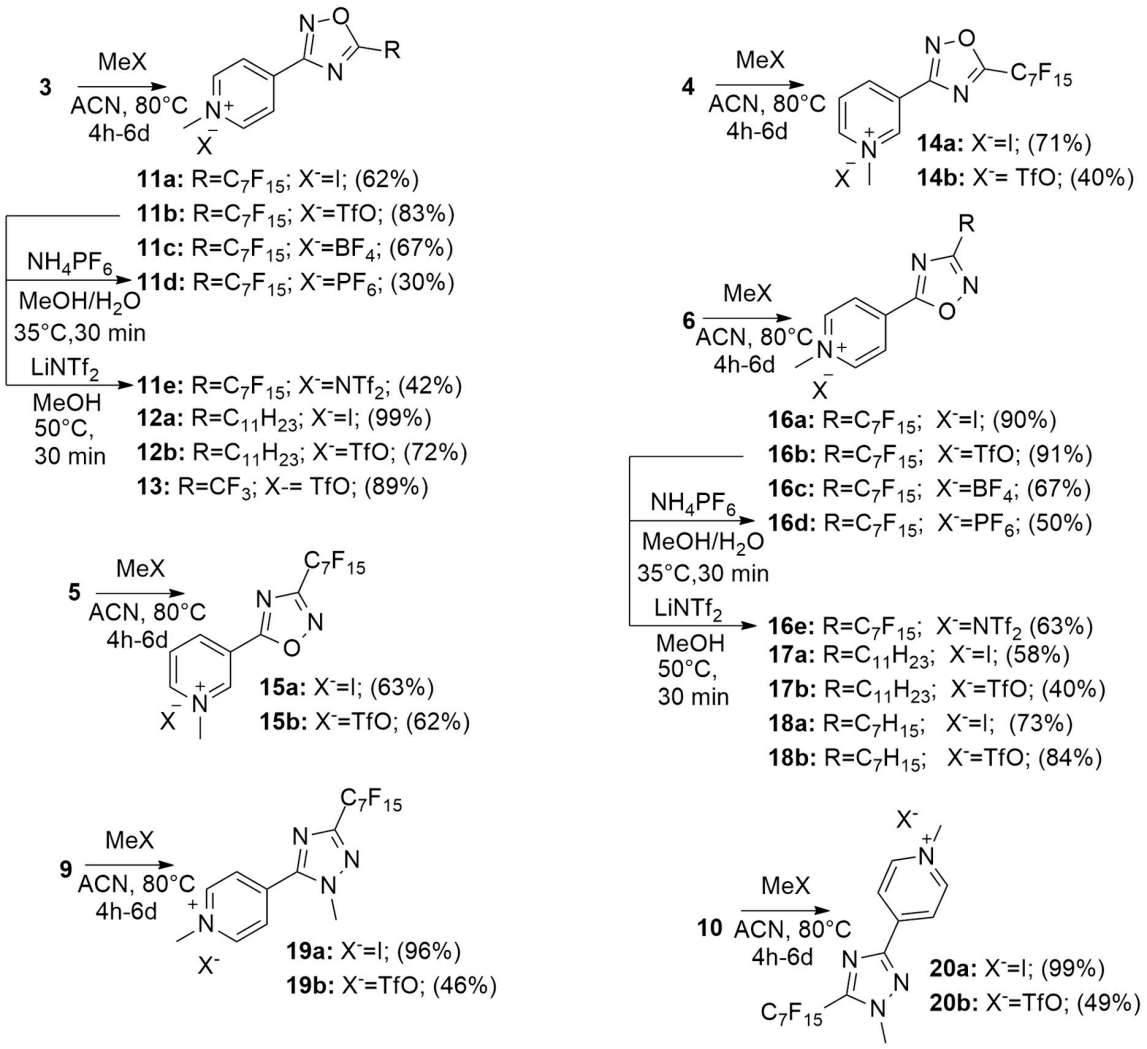
Synthesis of mono-cationic salts **11**–**20**.

The synthesis of the 3-alkyl-5-[4′-pyridyl]-1,2,4-oxadiazoles **6b**,**c** was performed through the reaction of amidoxime **8** with isonicotinoyl chloride **7** (Scheme 2). The triazole derivative was obtained through hydrazinolysis of **3a** with methylhydrazine. Both regioisomers were obtained, with 85% of the 1-methyl-3-perfluoroalkyltriazole (**9**) and 4% of the 1-methyl-5-perfluoroalkyltriazole (**10**), respectively (Scheme 2). To obtain the salts **11**–**20**, a quaternization reaction was performed with the appropriate methylating reagents, i.e., methyl iodide, methyl trifluoromethanesulfonate, or trimethyloxonium tetrafluoroborate (Scheme 3). Hexafluorophosphate and bis(trifluoromethanesulfonimide) salts were synthesized through metathesis processes (Scheme 3).

**Scheme 4:**
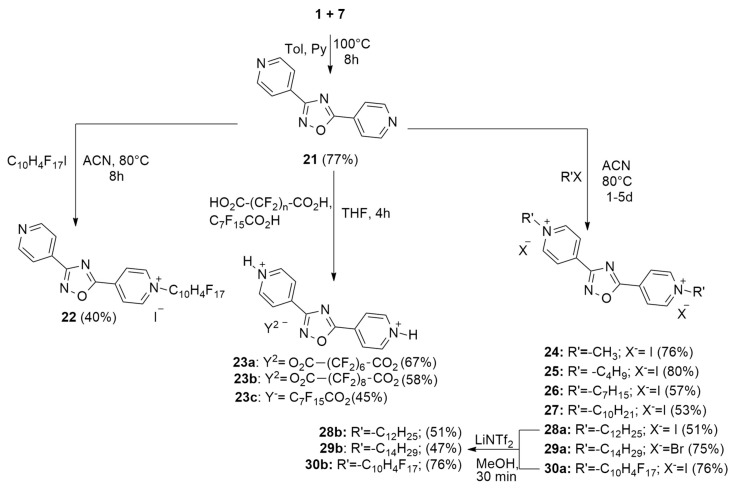
Synthesis of bis-pyridinium salts **22**–**30**.

A dipyridine with an oxadiazole spacer, the **21** core, was obtained through the reaction of isonicotinoyl amidoxime **1** and isonicotinoyl chloride **7** (Scheme 4). Starting from **21**, a set of mono- and di-cationic salts was obtained. In particular, the mono-cationic salt **22** was obtained from the reaction of bis-pyridinium compound **21** with the heptadecafluoro iododecane in a 1:1 ratio to yield the monopolyfluoroalkyl derivate. The di-cationic iodide and bromide salts (**24**–**27**, **28a**–**30a**) were prepared through a bis-alkylation or polyfluoroalkylation on **21**. On the other hand, the bistriflimide derivates (**28b**–**30b**) were converted from the corresponding **28a**, **29a**, and **30a** through the anion metathesis reaction [28] (Scheme 4). The salts **23a**–**c** were synthetized from **21** by combining the suitable perfluoroalkyl-carboxylic acid (1:1 or 1:2 ratio) (Scheme 4).

All synthesized compounds were characterized by means of NMR and HRMS. The obtained results (see Figures S1–S8) are in good agreement with the theoretical data and the literature. Concerning the structural characterization of quaternary pyridinium cations, it is worth noting the presence of the signal of the alkyl group directly linked to the positively charged nitrogen (around 4.1–4.8 ppm), which confirms alkylation of the pyridine ring. HRMS spectra performed by means of HPLC/MS/ESI in both polarities confirmed the molecular formula of cations as well as anion exchange after metathesis reactions.

### 2.2. Antibacterial Activity

**Figure 1:**
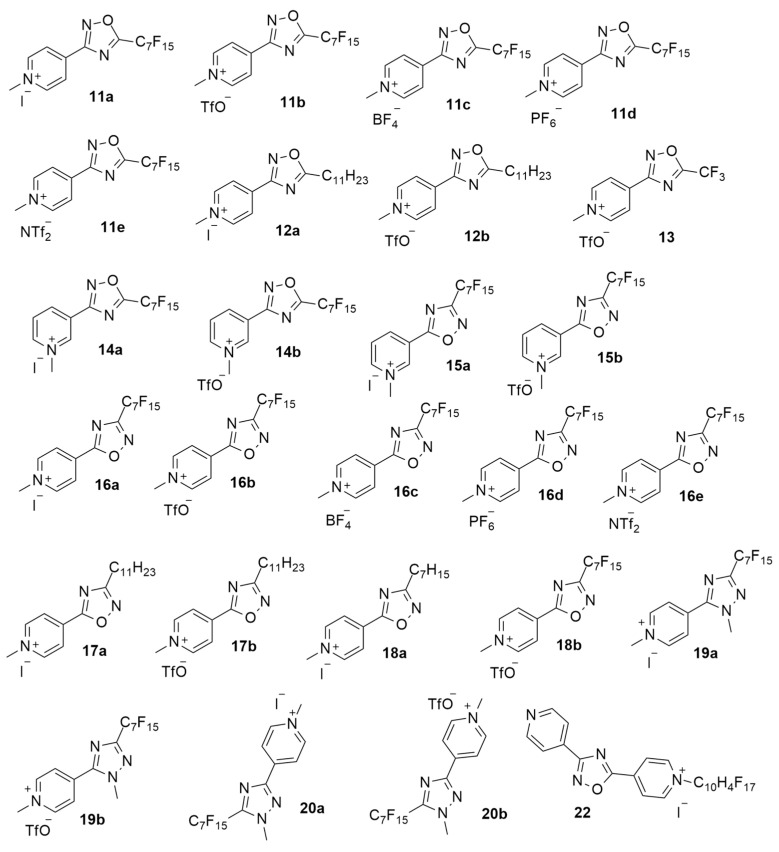
Mono-cationic salts.

The antibacterial activity of synthesized salts and reference drugs was tested, and Minimum Inhibitory Concentration (MIC) values of the mono-cationic pyridinium salts (see Figure 1) against reference strains of *S. aureus* ATCC (American Type Culture Collection) and *E. coli* ATCC have been reported in Table 1. Compounds from **11** to **22** showed very low or null (MIC ≥ 64 µg/mL) antibacterial activity against the Gram-negative strain of *E. coli*. On the contrary, significant shifts in MIC values against Gram-positive strains were observed.

Compounds **11**–**14** contain an 1,2,4-oxadiazole core with the pyridine ring substitution at position C-3 and the (perfluoro)alkyl chain at position C-5 of the heterocycle. Among these, salts **14a**,**b**, bearing a 3′-methyl-pyridinium moiety are inactive as well as compound **13** with a short CF_3_ side chain. Compounds **11** and **12** bearing a 4′-methyl-pyridinium moiety, show higher activity, in particular, the unfluorinated derivatives **12a**,**b** (MIC = 2 µg/mL).

The regioisomers **15**–**18** are characterized by a 1,2,4-oxadiazole core with the pyridine ring at C-5 and the side chain at C-3 of the oxadiazole. The sub-set of **15a**,**b** appears to be less active than the rest of this group (MIC > 64 µg/mL), this can be attributed to the presence of the 3′-methylpyridinium group at C-3, as previously observed. Derivatives **16a**–**d** with a perfluoroheptyl side-chain at C-3, are moderately potent (MIC = 8 µg/mL) and present higher activity than their regioisomer **11a**–**d**. Also, in this set of salts, those with a C_11_ alkyl chain are particularly active with MIC values of 1 µg/mL for the iodide **17a** and 2 µg/mL for the methyltriflate **17b**. Interestingly, for compounds **18a**,**b** the shortening of the alkyl chain to C_7_ causes the loss of activity. Changing the heterocyclic core from a 1,2,4-oxadiazole to a methyl-1,2,4-triazole (**19a**,**b**–**20a**,**b**) did not affect the antimicrobial activity. Compound **22** with a mono- alkylated bi-pyridyl-1,2,4-oxadiazole core present a good activity (MIC = 2 µg/mL), considerably better than the corresponding bis-alkylated derivatives **30a**,**b** (see Table 2). 

**Figure 2:**
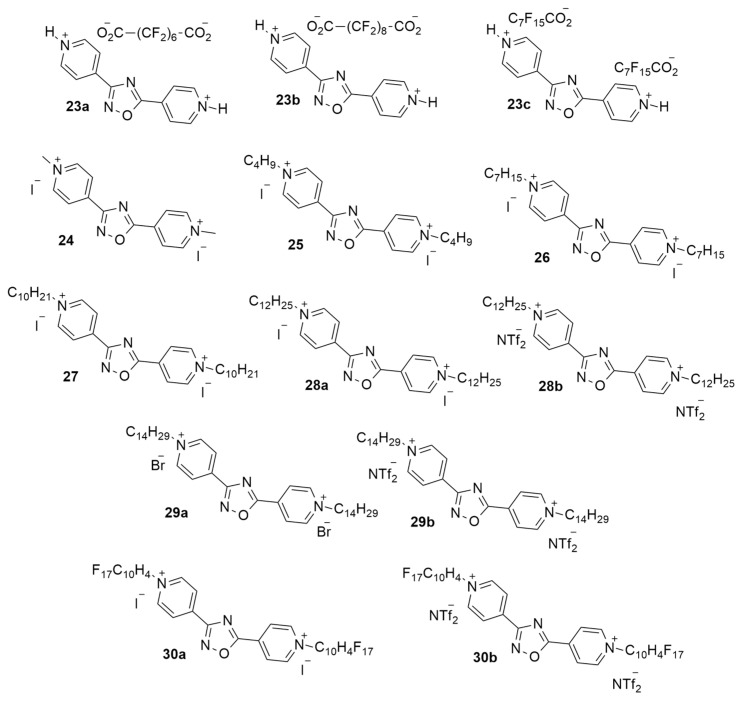
Bi-cationic salts.

MIC values against reference strain of *S. aureus* and *E. coli* of the bi-cationic pyridinium salts (see Figure 2) are reported on Table 2. These gemini quaternary ammonium compounds are characterized by two pyridinium heads connected through the 1,2,4-oxadiazole core.

Compounds **23**–**29** differ in the side chain’s length and also for the anions. Notably, the salts with an alkyl chain greater than, or equal to, C_10_ have interesting antibacterial activity. Compounds **27** and **28a**,**b** showed good activity against the *S. aureus* strain with an MIC = 0.25 µg/mL. Interestingly, compound **27** displayed an excellent MIC value (0.5 µg/mL) against the Gram-negative reference strain. Notably, for compound **29**, increasing the chain length to C_14_ reduced the antibacterial activity against the Gram-positive strain and caused activity loss against the Gram-negative representative strain. If we focus on the different contributions of the counterion, the bromide salt **29a** has a lower MIC value compared to the bistriflimide derivate **29b**.

From the data collected in Table 1 and Table 2, some interesting structure–activity relationships are evident. For example, the regioisomer containing a 3′-methyl-pyridinium moiety are less active than the corresponding 4′-derivatives. The 1,2,4-oxadiazole isomers with the chain linked at C-3 are more potent than the regio-isomer with a C-5 linked chain. Considering the side-chain effects, compounds presenting a longer chain are more active; in particular, the activity order is C_11_H_23_ > C_7_F_15_ > C_7_H_15_ because hydrophobic chain length is one of the most crucial factors in the structure of lipids, which, combined with the different polar heads, changes the shape of amphiphilic molecules and the chemical mechanism of contact with membranes. The role of the anion does not seem particularly relevant, and halides could be preferred for their synthetic feasibility. Concerning di-cationic compounds, their activity against Gram-negative bacteria is of great interest and is clearly influenced by the length of the alkyl chains.

Among all of the 39 salts, 4 mono-cationic (**12a**,**b**, **17a**,**b**) and 2 bi-cationic (**27** and **28b**) compounds were tested on isolated clinical strains of the Gram-positive bacteria *S. aureus*, *S. epidermidis*, and *S. haemolyticus* (Table 3).

Di-cationic 1,2,4-oxadiazole **27** and **28b** derivatives have shown better antibacterial activity against the seven Gram-positive, isolated strains with respect to the mono-cationic derivatives. In the group of the mono-cationic salts, **17a** and **17b** were selected to be investigated for further analysis. 

At the same time, compound **27** was tested on Gram-negative, isolated strains of *K. pneumoniae, A. baumannii,* and *P. aeruginosa*, presenting moderate antimicrobial action against the Gram-negative, resistant strains (Table 4).

Furthermore, the biofilm-forming capacity was analysed in treated reference strains, *S. aureus* and *E.coli,* and in two clinical isolates, *S.haemolyticus* 32076211 and *A.baumannii* 32111798, through the crystal violet method. The tests revealed a moderate to low biofilm production for Gram-positive and Gram-negative bacteria in the presence of **27**, **28a,** and **17a** compounds, which inhibit biofilm formation at sub-MIC concentrations. The obtained values range from 22.0 to 97.4% (Table 5).

A SwissADME simulation on the most active compounds, **12a**–**b**, **17a**–**b**, **27**, and **28a**, was performed in order to hypothesize the bioavailability and the type of application of these salts (see Figures S9–S14). [35] The obtained compounds present a good predicted profile; in particular, the mono-cationic derivative does not present a violation of the Lipinsky rules, while bi-cationic derivatives present MW > 500 and high MLOGP. 

## 3. Material and Methods

### 3.1. Materials

Trimethyloxonium tetrafluoroborate, ammonium hexafluorophosphate, bis(trifluoromethane)sulfonimide lithium, methyl trifluoromethanesulfonate, 1-iodoheptane, magnesium sulfate ethyl acetate, petroleum ether, dicloromethane, methanol, water, and acetonitrile were purchased from a commercial source and used as received. Column chromatography on silica gel, technical grade, pore size 60 Å, 230–400 mesh, and particle size 40–63 μm and TLC silica gel 60 F_254_ on aluminium support were used. Compounds **3a**–**c**, **4**, **5**, **6a**–**c**, **9**, **10**, **11a**–**b**, **12a**–**b**, **14a**–**b**, **15a**–**b**, **16a**–**b**, **17a**–**b**, **18a**–**b**, **19a**–**b**, **20a**–**b**, **21**, **22**, **23a**–**c**, **24**, **25**, **27**, **28a**–**b**, **29a**–**b**, and **30a**–**b** synthesis and characterization are in agreement with the previously reported data (see Supplementary Material) [26,27,28,29,30,31,32,36,37].

### 3.2. Methods

^1^H-NMR spectra were recorded on Bruker 300 MHz or 250 MHz spectrometers. The residual solvent peak was used as the reference. Melting points were determined on a REICHART-THERMOVAR hot-stage apparatus. Reversed-phase HPLC/ESI/Q-TOF HRMS experiments were performed using mixtures of water and acetonitrile of HPLC/MS grade as eluents with the addition of 0.1% (*v*/*v*) of formic acid. The HPLC system was an Agilent 1260 Infinity. A reversed-phase C18 column (Luna Omega 5 µm Polar C18 150 × 2.1 mm) with a Phenomenex C18 security guard column (4 mm × 3 mm) was used. The flow rate was 1 mL/min, and the column was set to 40 °C. The eluent was varied, and its composition was changed with a linear gradient. Initially, for 10 min, the linear gradient passed from 95% to 5% water; for a further 10 min, the gradient was inverted and passed from 5% to 95%; water and, for the last 5 min, it turned to a gradient of 95% to 5% water. The volume injected was 10 µL. MS TIC and UV (250 nm) were used to monitor the eluate. Mass spectra were registered on an Agilent 6540 UHD accurate-mass Q-TOF spectrometer equipped with a Dual AJS ESI source working in positive or negative mode. N_2_ was used as a desolvation gas at 300 °C and a flow rate of 9 L/min. The nebulizer was set to 45 psi. The Sheath gas temperature was set at 350 °C and a flow of 12 L/min. A potential of 3.5 kV or 2.6 kV was used on the capillary for positive or negative ion mode, respectively. The fragmentor was set to 110 V. MS spectra were recorded in the 150–2000 *m*/*z* range.

### 3.3. Synthesis and Characterization

#### 3.3.1. General Procedure for the Synthesis of Compounds **11c** and **16c**

The oxadiazoles **3a** or **6a** (1 mmol) were dissolved in DCM (5–8 mL) in an ACE pressure tube and (CH_3_)_3_OBF_4_ (20 mol eq.) was added. The mixture was stirred and heated at 60 °C for 4–6 days. The reaction was monitored using TLC (petroleum ether and ethyl acetate 5:1 and 1:1). After cooling, the mixture was dried, water (50 mL) was added, the pH was adjusted to neutral, and the organic compounds were extracted with ethyl acetate (30 mL ×3). The organic phase was dried with MgSO_4_, and the compounds **11c**–**16c** were isolated through chromatography (petroleum ether/ethyl acetate 5:1 and 1:1).

***N*-Methyl-4-(5-pentadecafluoroheptyl-1,2,4-oxadiazol-3-yl)pyridinium tetrafluoroborate (11c)**

Yield = 67%, yellow solid, m.p. = 120 °C, ^1^H NMR (400 MHz, DMSO) δ 9.23 (d, *J* = 6.6 Hz, 2H), 8.74 (d, *J* = 6.6 Hz, 2H), 4.46 (s, 3H); ESI-MS analysis for [C_15_H_7_F_15_N_3_O^+^]: Calc.: 530.0344 *m*/*z*, exp.: 530.0355 *m*/*z*; for [BF_4_^−^]: Calc.: 87.0035 *m*/*z*, exp.: 87.0036 *m*/*z*.

***N*-Methyl-4-(3-pentadecafluoroheptyl-1,2,4-oxadiazol-5-yl)pyridinium tetrafluoroborate (16c)**

Yield= 67%, yellow solid, m.p. = 100 °C, ^1^H NMR (400 MHz, DMSO) δ 9.26 (d, *J* = 6.8 Hz, 2H), 8.82 (d, *J* = 6.8 Hz, 2H), 4.47 (s, 3H); ESI-MS analysis for [C_15_H_7_F_15_N_3_O^+^]: Calc.: 530.0344 *m*/*z*, exp.: 530.0344 *m*/*z*, for [BF_4_^−^]: Calc.: 87.0035 *m*/*z*, exp.: 87.0034 *m*/*z*.

#### 3.3.2. General Procedure for the Synthesis of Compounds **11d** and **16d**

Compounds **11a** or **16a** (1 mmol) were dissolved in a minimum amount of MeOH (3–10 mL) and NH_4_PF_6_ (5 mol eq.) was added. The mixture was heated up to 30–35 °C for 0.5 h. Then, water was added dropwise until a precipitate was formed. The mixture was stirred for 1 h. The solid was filtered under vacuum and washed with water.

***N*-Methyl-4-(5-pentadecafluoroheptyl-1,2,4-oxadiazol-3-yl)pyridinium hexafluorophosphate (11d)**

Yield = 30%, yellow solid, m.p. 98 °C, ^1^H NMR (400 MHz, DMSO) δ 9.21 (d, *J* = 6.7 Hz, 2H), 8.73 (d, *J* = 6.7 Hz, 2H), 4.45 (s, 3H); ESI-MS analysis for [C_15_H_7_F_15_N_3_O^+^]: Calc.: 530.0344 *m*/*z*, exp.: 530.0353 *m*/*z*, [PF_6_^−^]: Calc.: 144.9647 *m*/*z*, exp.: 144.9648 *m*/*z*.

***N*-Methyl-4-(3-pentadecafluoroheptyl-1,2,4-oxadiazol-5-yl)pyridinium hexafluorophosphate (16d)**

Yield = 50%, yellow solid, m.p. 115 °C, ^1^H NMR (400 MHz, DMSO) δ 9.28 (d, *J* = 6.8 Hz, 2H), 8.84 (d, *J* = 6.8 Hz, 2H), 4.48 (s, 3H); ESI-MS analysis for [C_15_H_7_F_15_N_3_O^+^]: Calc.: 530.0344 *m*/*z*, exp.: 530.0345 *m*/*z*, [PF_6_^−^]: Calc.: 144.9647 *m*/*z*, exp.: 144.9648 *m*/*z*.

#### 3.3.3. General Procedure for the Synthesis of Compounds **11e** and **16e**

The oxadiazoles salts (**11b** or **16b**, 1 mmol) were dissolved in the minimal amount MeOH (3–10 mL) in a glass vessel and LiNTf_2_ (2.5 mol eq.) was added. The mixture was stirred for 0.5 h at 50 °C, and then water was added dropwise until a permanent suspension was formed. The mixture was stirred for a further 2 h and the solid was filtrated under vacuum, washed with water, and dried.

***N*-Methyl-4-(5-pentadecafluoroheptyl-1,2,4-oxadiazol-3-yl)pyridinium bis((trifluoromethyl)sulfonyl)amide (11e)**

Yield = 42%, white solid, m.p. = 58 °C, ^1^H NMR (400 MHz, DMSO) δ 9.23 (d, *J* = 6.6 Hz, 2H), 8.74 (d, *J* = 6.6 Hz, 2H), 4.46 (s, 3H); ESI-MS analysis for [C_15_H_7_F_15_N_3_O^+^]: Calc.: 530.0344 *m*/*z*, exp.: 530.0347 *m*/*z*, [NTf_2_^−^]: Calc.: 279.9178 *m*/*z*, exp.: 279.9184 *m*/*z*.

***N*-Methyl-4-(3-pentadecafluoroheptyl-1,2,4-oxadiazol-5-yl)pyridinium bis((trifluoromethyl)sulfonyl)amide (16e)**

Yield = 63%, white solid, m.p. = 80 °C, ^1^H NMR (400 MHz, DMSO) δ 9.27 (d, *J* = 6.8 Hz, 2H), 8.83 (d, *J* = 6.8 Hz, 2H), 4.47 (s, 3H); ESI-MS analysis for [C_15_H_7_F_15_N_3_O^+^]: Calc.: 530.0344 *m*/*z*, exp.: 530.0345 *m*/*z*, [NTf_2_^−^]: Calc.: 279.9178 *m*/*z*, exp.: 279.9178 *m*/*z*.

#### 3.3.4. Synthesis of 1-Methyl-4-(5-(trifluoromethyl)-1,2,4-oxadiazol-3-yl)pyridin-1-ium trifluoromethanesulfonate (**13**)

The 3-(pyridin-4-yl)-5-(trifluoromethyl)-1,2,4-oxadiazole (**3c**) (200 mg, 0.4 mmol) was dissolved in 20 mL of anhydrous acetonitrile, and then the methyl trifluoromethanesulfonate (730 mg, 4.4 mmol) was added to the mixture. The reaction was allowed to stir at room temperature overnight. The solvent was removed under reduced pressure and the residue was purified through column chromatography on silica gel with ethyl acetate and then with ACN.

Yield = 89%, white solid, m.p. = 76 °C, ^1^H NMR (300 MHz, CD_3_CN) δ (ppm) = 8.9 (d, *J* = 8.1 Hz, 2H), 8.61 (d, *J* = 8.1 Hz, 2H), 4.42 (s, 3H); ESI-MS analysis for [C_9_H_7_F_3_N_3_O^+^]: Calc.: 230.0536 *m*/*z*, exp.: 230.0536 *m*/*z*, [TfO^−^]: Calc.: 148.9526 *m*/*z*, exp.: 148.9527 *m*/*z*.

#### 3.3.5. Synthesis of 4,4’-(1,2,4-Oxadiazole-3,5-diyl)bis(1-heptylpyridin-1-ium) iodide (**26**)

The 3,5-Di(pyridin-4-yl)-1,2,4-oxadiazole **21** (0.5 g, 2.230 mmol) was dissolved in canACN (16 mL) in an ACE pressure tube and 1-iodoheptane (40 mol eq.) was added. The solution was refluxed under stirring at 80 °C for 5 days, after which the solvent was filtered under vacuum. The residue was recovered after washing with cold chloroform.

Yield = 57%, brown solid, ^1^H NMR (400 MHz, DMSO-d_6_) δ 9.44 (d, *J* = 6.8 Hz, 2H), 9.37 (d, *J* = 6.9 Hz, 2H), 8.89 (d, *J* = 6.9 Hz, 2H), 8.78 (d, *J* = 6.8 Hz, 2H), 4.74 (m, 4H), 1.98 (s, 4 H), 1.30 (m, 16H), 0.86 (m, 6H); ESI-MS analysis for [C_26_H_38_N_4_O_2_^2+^]: Calc.: 211.1517 *m*/*z*, exp.: 211.1517 *m*/*z*.

### 3.4. Antimicrobial Activity According to Minimum Inhibitory Concentrations (MICs) Determination

The antibacterial activities of compounds **11** to **30** were evaluated in vitro against reference strains *S. aureus* (ATCC 25923) and *E. coli* (ATCC 25922) and toward fourteen clinical strains isolated using central venous catheters (seven Gram-positive bacteria, including three *S. aureus*, two *S. epidermidis*, and two *S. haemolyticus,* and seven Gram-negative bacteria, including five *Klebsiella pneumoniae*, one *Acinetobacter baumanii*, and one *Pseudomonas aeruginosa,* respectively).

The reference and clinical strains were tested with different classes of antibiotics according to the EUCAST guidelines using the microdilution method. Except for *E. coli* ATCC 25922, all Gram-negative clinical strains were multidrug-resistant (MDR), extended spectrum beta-lactamase (ESBL), and carbapenemase-producing bacteria. Antimicrobial susceptibility testing was performed for penicillins, cephalosporins, carbapenems, fluoroquinolones, and aminoglycosides. For Staphylococcus strains, antibiotics tested belonged to penicillins, fluoroquinolones, aminoglycosides, glicopeptides, macrolides, tetacyclines, and oxazolidinones. All strains, except for *S. aureus* ATCC 25923, were resistance to oxacillina and, in a variable way, to other molecules. *S. haemolyticus* strains were those that had resistance to multiple drug classes.

MICs were determined through the broth microdilution method according to the European Committee on Antimicrobial Susceptibility Testing (EUCAST) guidelines (https://www.eucast.org/ast_of_bacteria (accessed on 20 December 2023)). 

Briefly, bacterial inocula were prepared by taking individual colonies of each strain from cultured plates and resuspending in sterile saline to obtain turbidity equivalent to a 0.5 McFarland standard (1.5 × 10^8^ CFU). The suspensions were diluted in Mueller–Hinton broth (Sigma-Aldrich, St-Louis, MO, USA) to a final bacterial cell density of 5 × 10^5^ CFU/ mL and plated in sterile 96-well microtiter plate. The compound dilution range chosen was 0.125–64 mg/L according to a 1:2 dilution series on a final well volume of 100 μL, and each antimicrobial assay was performed in triplicate.

After 18–20 h of incubation at 35–37 °C, the MIC was defined as the lowest concentration of substance at which there was no visible growth. 

### 3.5. Biofilm Inhibition Assay

The inhibition of biofilm formation was assessed using the crystal violet method according to the protocol described by O’ Toole et al. (2000) [38,39]. Bacterial suspensions (5 × 10^5^ cfu/mL) were added to a 96-well polystyrene microtiter plate (Sigma Aldrich) with MH broth as the control for biofilm formation and with the sub-MIC concentration of the compounds for biofilm inhibition. The plates were incubated overnight at 37 °C under static conditions to allow for bacterial growth and biofilm maturation. After incubation, the microplates were shaken to discard planktonic cells, and adherent bacteria were rinsed after three times with distilled water. The biomass stained with 0.1% CV solution was finally quantified by recording the Optical density (OD) with a wavelength of 540 nm of each well using a microtiter plate reader (Labsystems Multiskan^®^ MCC/340). Assays were performed in triplicate and repeated at least three times. The bacterial strains were considered biofilm producers when their OD values were 3 times greater than standard deviation of the mean OD value of negative controls (ODNC). The biofilm-positive phenotypes were classified as weak (ODNC < OD ≤ 2 × ODNC), moderate (2 × ODNC < OD ≤ 4 × ODNC), or strong (OD > 4 × ODNC) biofilm producers, according to Stepanovi [39]. The well-characterized biofilm-producing strain *S. epidermidis* ATCC 35984 and the biofilm-negative *Staphylococcus aureus* ATCC 25923 strain were used as positive and negative controls, respectively. The percentages of biofilm inhibition were calculated using the following formula:


$$\%\;of\;inhibition=\frac{ODgrowht\;control-ODsample}{ODgrowth\;control}$$

## 4. Conclusions

A series of antibacterial salts varying in charged head, heterocyclic core, length of alkyl chain, and anion has been synthesized. The ability of these mono- and bi-cationic pyridine salts to counteract the growth of resistant bacteria was evaluated through determination of MIC against ATCC and clinically isolated strains. An initial analysis shows that isomers **12a**,**b** and **17a**,**b** are the most active against Gram-positive bacteria.

From preliminary assays, the bi-cationic compounds **27** and **28a**,**b** already showed higher inhibition data compared to the methyl pyridinium salts. In fact, they all have MIC values < 2 μg/mL against isolated strains of *S. aureus*, *S. epidermidis*, and *S. haemolyticus*, but they are also able to inhibit the growth of biofilms. The salt **28a** reaches an inhibition percentage of 94.1% against the resistant strain of *S. haemolyticus* 32076211, while salt **27** shows an inhibition percentage of 97.4% compared to the isolated strain *A. baumanni* 3211798. The side chain of carbon atoms of 10 and 12 carbon atoms is likewise the structural feature that distinguishes the salts mentioned in this case. The obtained compounds can be envisaged as sanitizers, and their application as antibiotics for systemic uses is under investigation. In fact, further optimization of the structure of this novel class of antibacterials is in due course.

## Figures and Tables

**Table 1:** Evaluation of antibacterial activity of mono-cationic pyridinium salts against reference strains of *S. aureus* and *E. coli*.

Compound	R	X^−^	MIC (µg/mL) *S. aureus* ^a^	MIC (µg/mL) *E. coli* ^b^
**11a**	C_7_F_15_	I	>64	>64
**11b**	C_7_F_15_	TfO	16	>64
**11c**	C_7_F_15_	BF_4_	16	>64
**11d**	C_7_F_15_	PF_6_	16	>64
**11e**	C_7_F_15_	NTf_2_	16	>64
**12a**	C_11_H_23_	I	2	>64
**12b**	C_11_H_23_	TfO	2	>64
**13**	CF_3_	TfO	>64	>64
**14a**	C_7_F_15_	I	>64	>64
**14b**	C_7_F_15_	TfO	>64	>64
**15a**	C_7_F_15_	I	>64	>64
**15b**	C_7_F_15_	TfO	>64	>64
**16a**	C_7_F_15_	I	8	>64
**16b**	C_7_F_15_	TfO	8	>64
**16c**	C_7_F_15_	BF_4_	8	>64
**16d**	C_7_F_15_	PF_6_	8	64
**16e**	C_7_F_15_	NTf_2_	16	>64
**17a**	C_11_H_23_	I	1	64
**17b**	C_11_H_23_	TfO	2	>64
**18a**	C_7_H_15_	I	>64	>64
**18b**	C_7_H_15_	TfO	>64	>64
**19a**	C_7_F_15_	I	8	>64
**19b**	C_7_F_15_	TfO	8	>64
**20a**	C_7_F_15_	I	8	>64
**20b**	C_7_F_15_	TfO	8	>64
**22**	C_10_H_4_F_17_	I	2	>64
**CIP**	-	-	≤0.5	-
**LZD**	-	-	1	-
**LVX**	-	-	-	≤0.5
**TZP**	-	-	-	≤4/4

^a^ ATCC 25923; ^b^ ATCC 25922. CIP = ciprofloxacin, LZD = linezolid, LVX = levofloxacin, TZP = piperacillin/tazobactam.

**Table 2:** Evaluation of antibacterial activity of di-cationic pyridinium salts against reference strains of *S. aureus* and *E. coli*.

Compound	R	X	MIC (µg/mL) *S. aureus* ^a^	MIC (µg/mL) *E. coli* ^b^
**23a**	H	O_2_C(CF_2_)_6_CO_2_	>64	>64
**23b**	H	O_2_C(CF_2_)_8_CO_2_	>64	>64
**23c**	H	C_7_F_15_CO_2_	>64	>64
**24**	CH_3_	I	>64	>64
**25**	C_4_H_9_	I	>64	>64
**26**	C_7_H_15_	I	>64	>64
**27**	C_10_H_21_	I	0.25	0.5
**28a**	C_12_H_25_	I	0.25	4
**28b**	C_12_H_25_	NTf_2_	0.25	4
**29a**	C_14_H_29_	Br	2	>64
**29b**	C_14_H_29_	NTf_2_	4	>64
**30a**	C_10_H_4_F_17_	I	>64	>64
**30b**	C_10_H_4_F_17_	NTf_2_	>64	>64
**CIP**	-	-	≤0.5	-
**LZD**	-	-	1	-
**LVX**	-	-	-	≤0.5
**TZP**	-	-	-	≤4/4

^a^ ATCC 25923; ^b^ ATCC 25922. CIP = ciprofloxacin, LZD = linezolid, LVX = levofloxacin, TZP = piperacillin/tazobactam.

**Table 3:** Evaluation of Minimum Inhibitory Concentration ^1^ on Gram-positive, resistant, isolated strains.

Strain	MIC 17a	MIC 17b	MIC 12b	MIC 12a	MIC 27	MIC 28a	MIC CIP	MIC LZD
*S. aureus* 32081873	8	8	8	8	2	2	>4	1
*S. aureus* 32106008	2	8	2	2	1	2	≤0.5	1
*S. aureus* 32116042	1	2	2	2	1	1	≤0.5	1
*S. epidermidis* 32080691	4	4	4	8	1	1	4	2
*S. epidermidis* 32078856	4	16	16	8	0.5	1	4	2
*S. haemolyticus* 32105436	8	16	16	16	1	1	>4	1
*S. haemolyticus* 32076211	8	8	8	8	0.5	1	>4	2

^1^ µg/mL. CIP = ciprofloxacin, LZD = linezolid.

**Table 4:** Evaluation of Minimum Inhibitory Concentration on Gram-negative, resistant, isolated strains.

Strain	MIC (µg/mL) 27	MIC (µg/mL) LVX	MIC (µg/mL) TZP
*K. pneumoniae* 32084798	16	>8	32/4
*K. pneumoniae* 32084853	16	>8	>32/4
*K. pneumoniae* 32086018	8	>8	>32/4
*K. pneumoniae* 32105351	8	>8	>32/4
*K. pneumoniae* 32108273	16	>8	32/4
*A. baumannii* 32111798	8	>8	>32/4
*P. aeruginosa* 32107321	8	1	8/4

LVX = levofloxacin, TZP = piperacillin/tazobactam.

**Table 5:** Biofilm inhibition.

	Biofilm Inhibition (%)		
Strain	27	28a	17a
*S. aureus* ATCC 25923	22.0	38.0	38.3
*S. haemolyticus* 32076211	44.3	94.1	29.5
*E. coli* ATCC 25922	48.9	48.8	N.D. ^a^
*A. baumannii* 32111798	97.4	39.5	N.D. ^a^

^a^ Not determined.

## Data Availability

New data were available on Supplementary Material.

## Supplementary Materials

The supporting information can be downloaded at: https://www.mdpi.com/article/10.3390/ijms25010377/s1.
