# Supplementary material for: Synthesis and Antibacterial Activity of Mono- and Bi-Cationic Pyridinium 1,2,4-Oxadiazoles and Triazoles
Source: Int J Mol Sci. 2023 Dec 27;25(1):377. doi: 10.3390/ijms25010377 (PMC10778652; doi:10.3390/ijms25010377)
Supplement: Supplementary file 1 [file ijms-25-00377-s001.zip › ijms-2776401-supplementary.pdf]

Supplementary Materials for

Synthesis and Antibacterial Activity of Mono- and Bi-Cationic Pyridinium 1,2,4-Oxadiazoles and Triazoles

Sara Amata [1,†], Cinzia Calà [2,3,†], Carla Rizzo [1,], Ivana Pibiri [1], Mariangela Pizzo [2], Silvestre Buscemi [1] and Antonio Palumbo Piccionello [1,*]

[1] Department of Biological, Chemical and Pharmaceutical Sciences and Technologies (STEBICEF), University of Palermo, Viale delle Scienze, Ed. 17, 90128 Palermo, Italy; sara.amata01@unipa.it (S.A.); carla.rizzo03@unipa.it (C.R.); ivana.pibiri@unipa.it (I.P.); silvestre.buscemi@unipa.it (S.B.)
[2] Department of Health Promotion, Mother and Child Care, Internal Medicine and Medical Specialties "G D'Alessandro", University of Palermo, Via Del Vespro 133, 90127 Palermo, Italy; cinzia.cala@unipa.it (C.C.); mariangela.pizzo22@gmail.com (M.P.)
[3] Microbiology and Virology Unit, AOU Policlinico "P. Giaccone", 90127 Palermo, Italy
* Correspondence: antonio.palumbopiccionello@unipa.it (A.P.P.)
† These authors contributed equally to this work.

Figure S1-S8. ^{1}H NMR, ESI (+), ESI (-) spectra of new compounds.
Figure S9-S14. Simulation of drug-like properties and ADME by SwissADME for most active compounds.

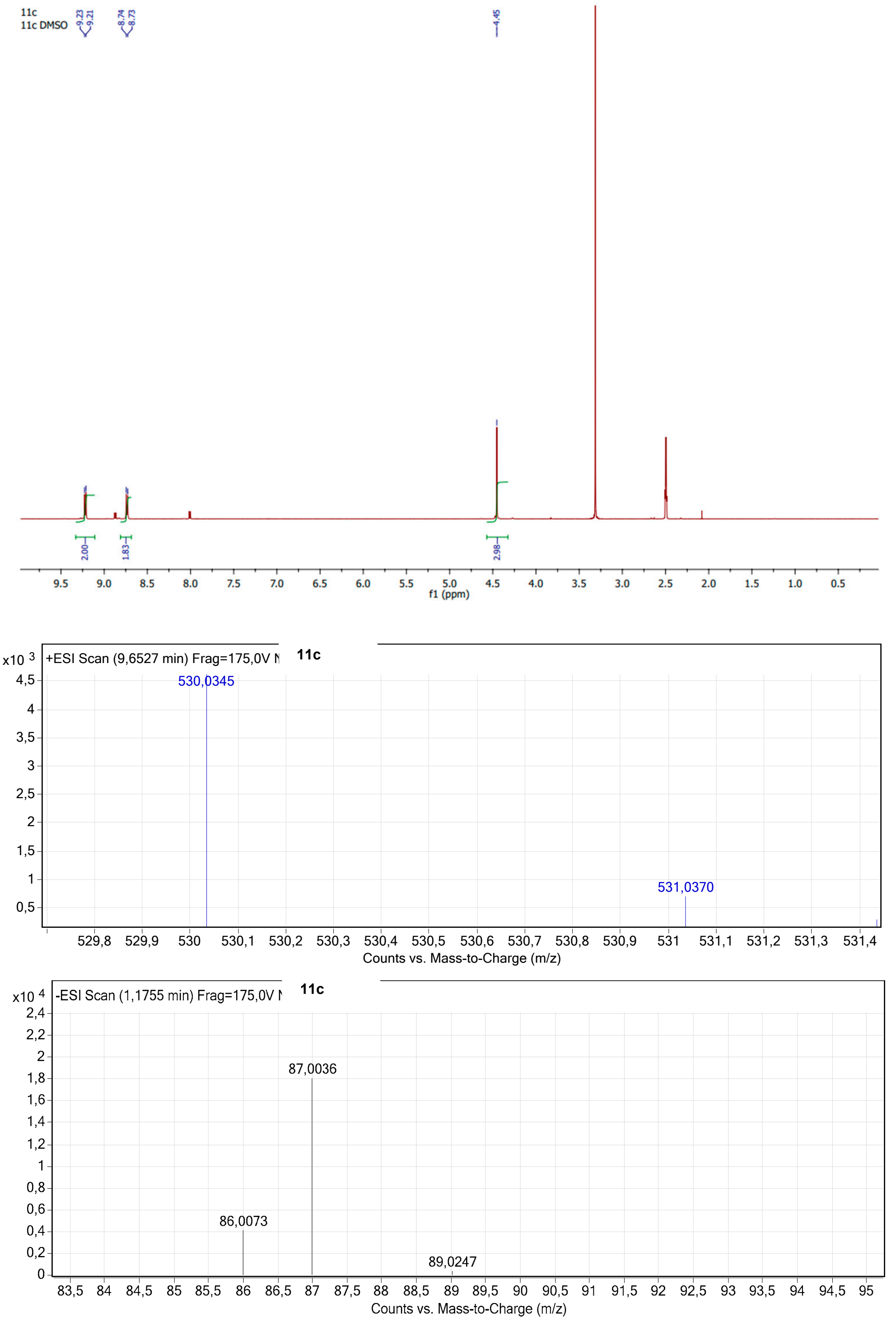

Figure S1. Spectrum of compound **11c,** ^{1}H NMR (400 MHz, DMSO-d_6) δ 9.22 (d, *J* = 6.6 Hz, 2H), 8.74 (d, *J* = 6.6 Hz, 2H), 4.45 (s, 3H). ESI-MS analysis for [$C_{15}H_7F_{15}N_3O^+$]: Calc.: 530.0344 m/z, exp.: 530.0345 m/z; for [BF_4^-]: Calc.: 87.0035 m/z, exp.: 87.0036 m/z.

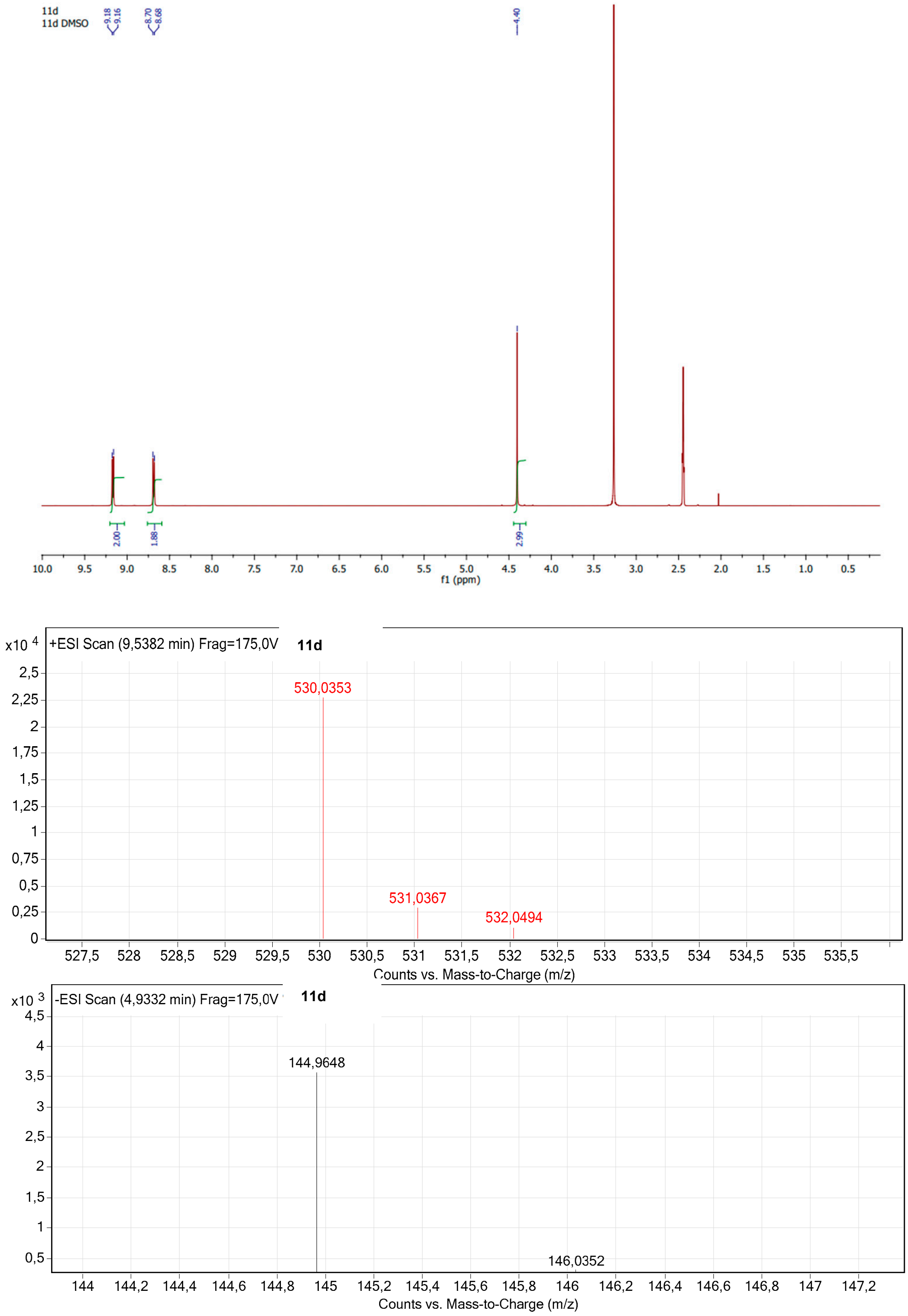

Figure S2. Spectrum of compound **11d,** ^{1}H NMR (400 MHz, DMSO-d$_6$) δ 9.17 (d, *J* = 6.7 Hz, 2H), 8.69 (d, *J* = 6.7 Hz, 2H), 4.40 (s, 3H). ESI-MS analysis for [$C_{15}H_7F_{15}N_3O^+$]: Calc.: 530.0344 m/z, exp.: 530.0353 m/z, [PF_6^-]: Calc.: 144.9647 m/z, exp.: 144.9648 m/z.

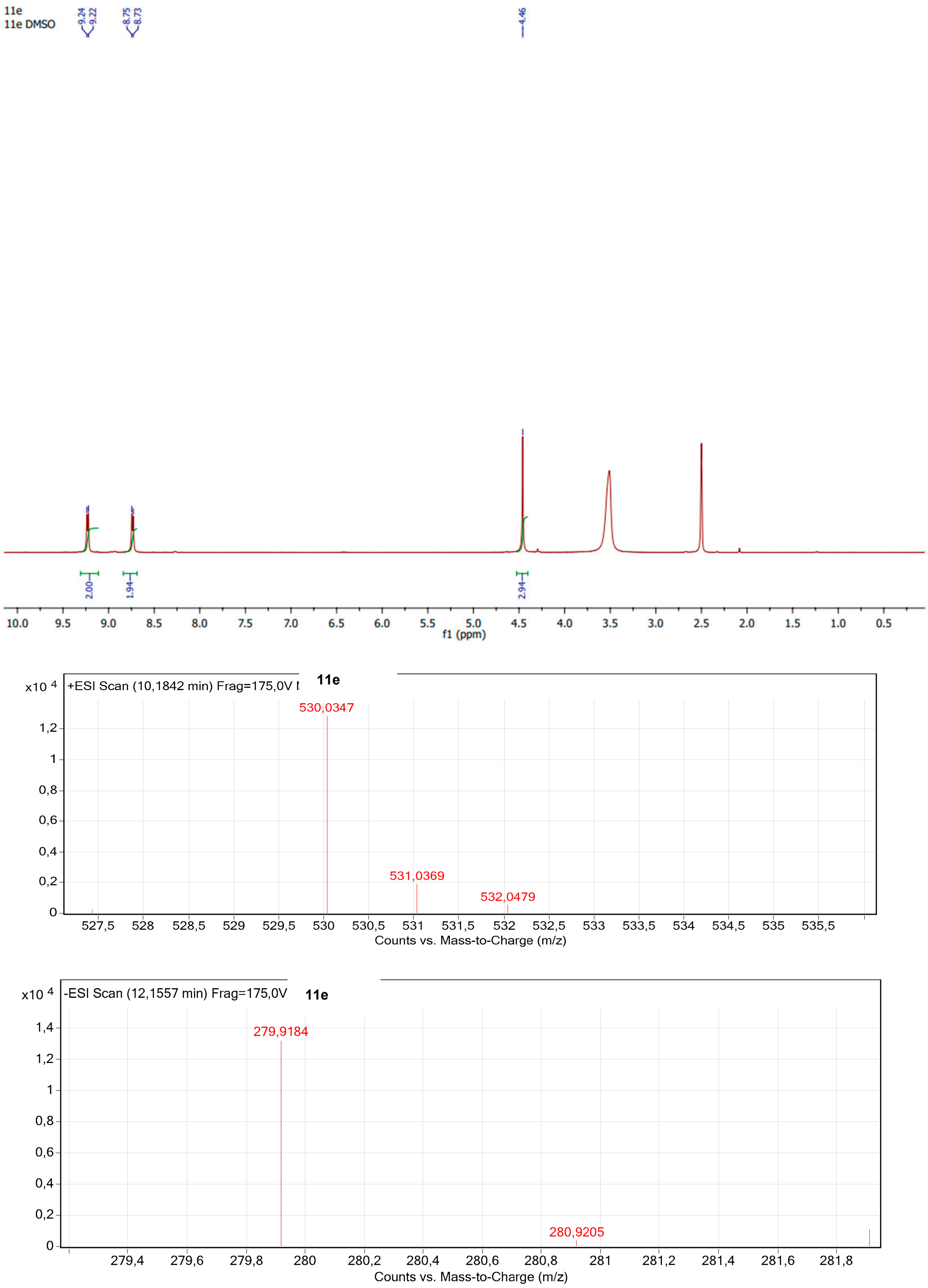

Figure S3. Spectrum of compound **11e,** ^{1}H NMR (400 MHz, DMSO-d$_6$) δ 9.23 (d, *J* = 6.6 Hz, 2H), 8.74 (d, *J* = 6.6 Hz, 2H), 4.46 (s, 3H). ESI-MS analysis for $[C_{15}H_7F_{15}N_3O^+]$: Calc.: 530.0344 m/z, exp.: 530.0347 m/z, $[NTf_2^-]$: Calc.: 279.9178 m/z, exp.: 279.9184 m/z.

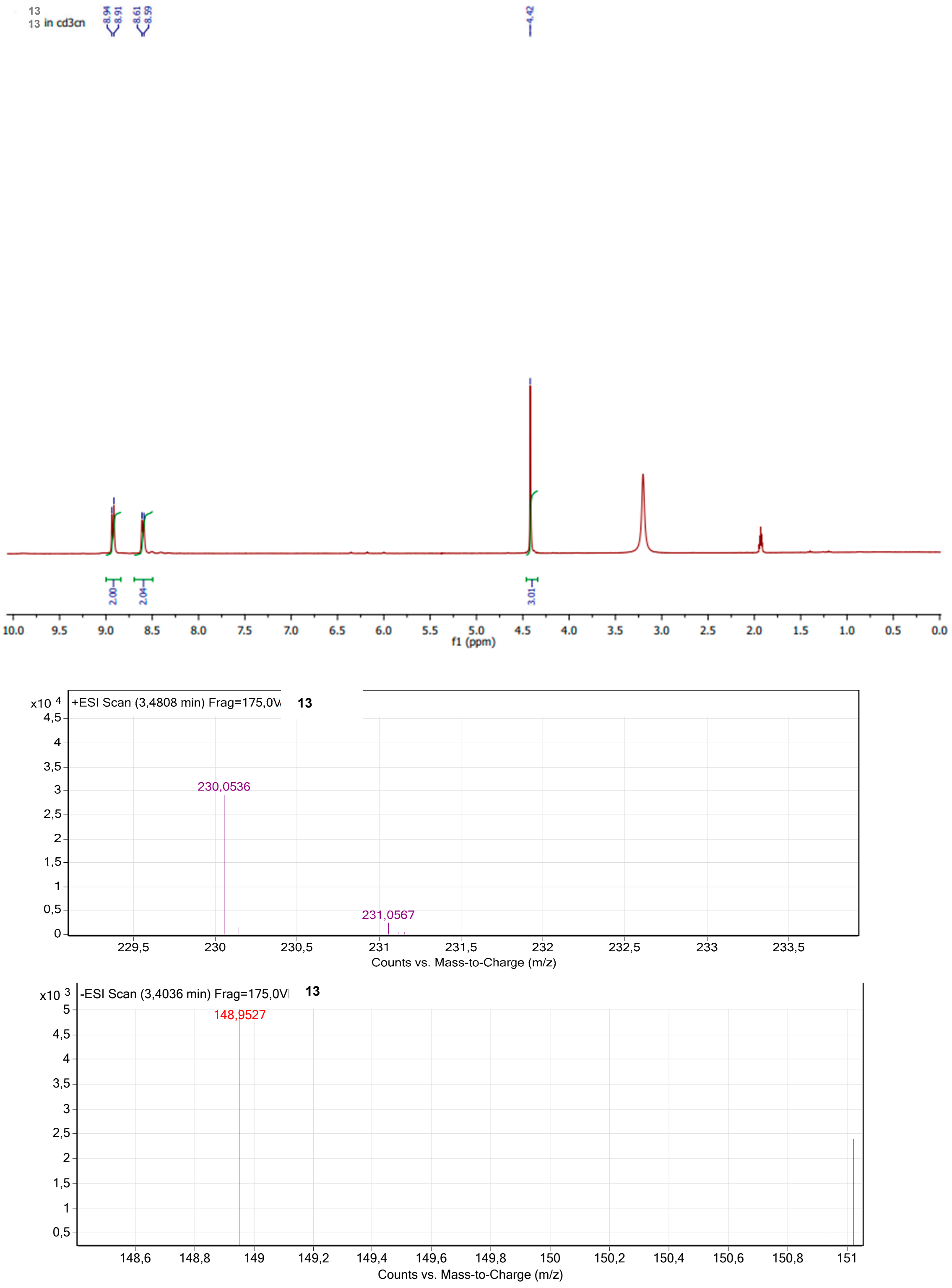

Figure S4. Spectrum of compound **13,** ^{1}H NMR (300 MHz, CD_3CN) δ (ppm) = 8.92 (d, *J*= 8.1 Hz, 2H), 8.60 (d, *J*= 8.1 Hz, 2H), 4.42 (s, 3H); ESI-MS analysis for [$C_9H_7F_3N_3O^+$]: Calc.: 230.0536 m/z, exp.: 230.0536 m/z, [TfO^-]: Calc.: 148.9526 m/z, exp.: 148.9527 m/z.

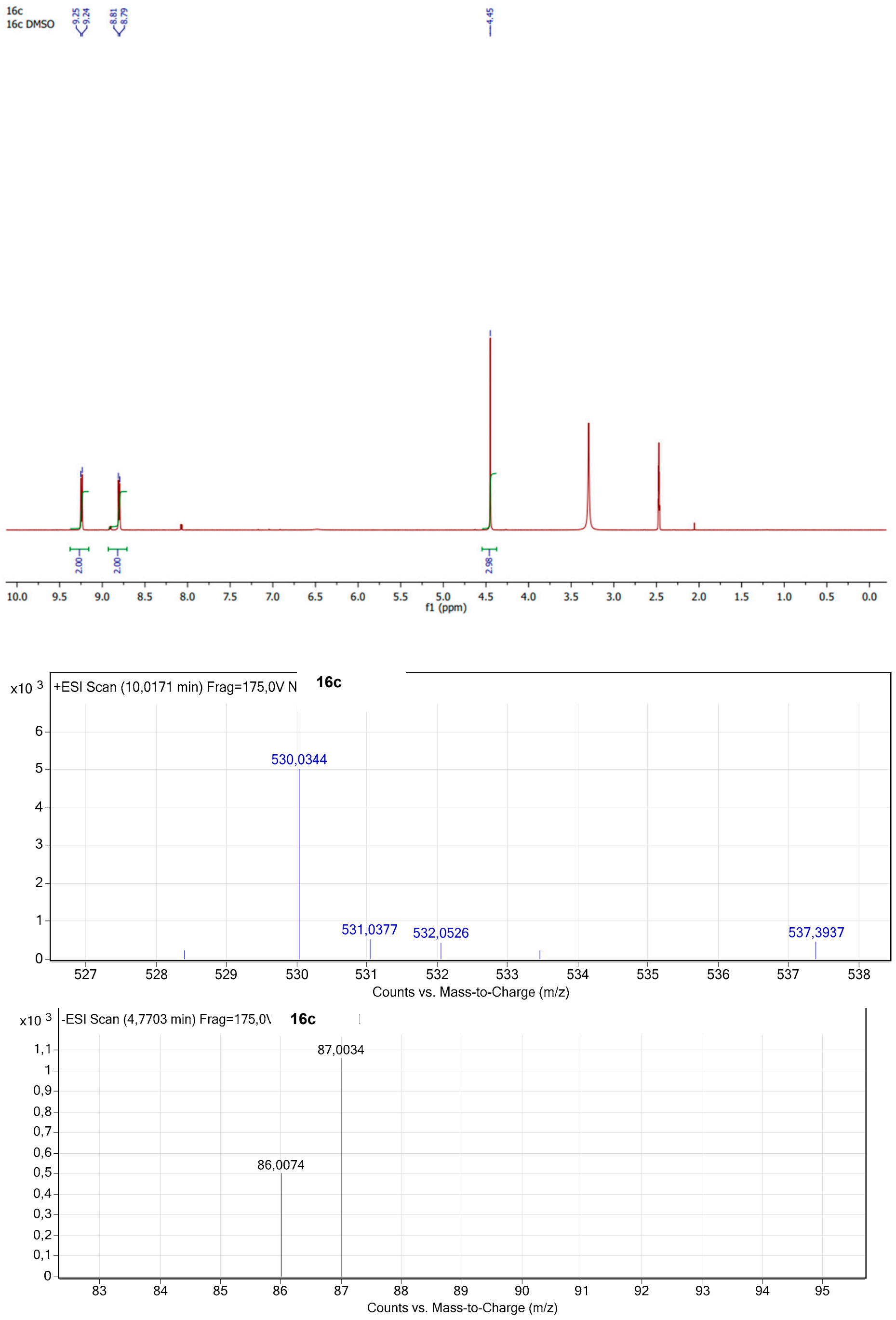

Figure S5. Spectrum of compound **16c,** ^{1}H NMR (400 MHz, DMSO-d_6) δ 9.25 (d, *J* = 6.8 Hz, 2H), 8.80 (d, *J* = 6.8 Hz, 2H), 4.45 (s, 3H). ESI-MS analysis for $[C_{15}H_7F_{15}N_3O^+]$: Calc.: 530.0344 m/z, exp.: 530.0344 m/z, for $[BF_4^-]$: Calc.: 87.0035 m/z, exp.: 87.0035 m/z.

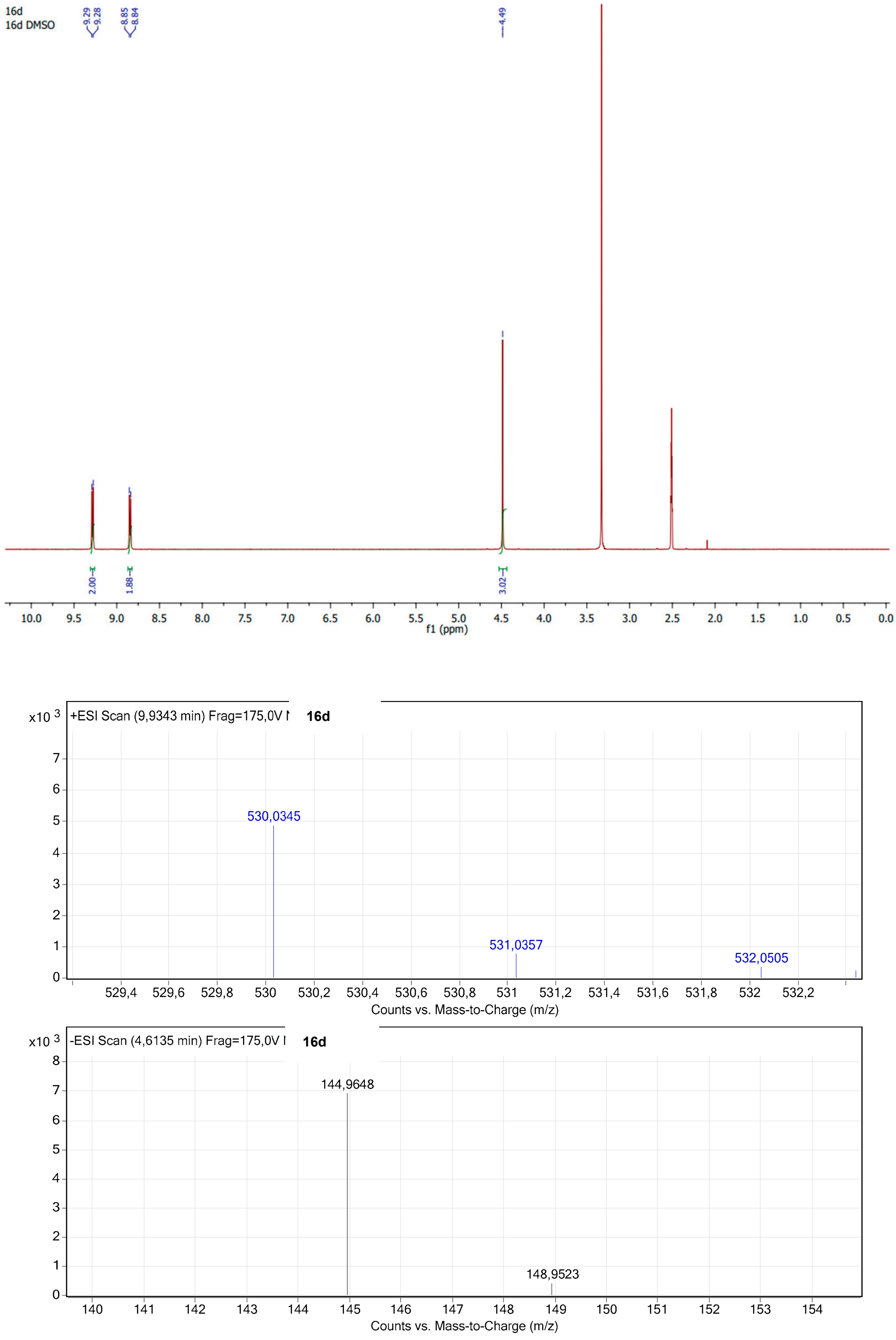

Figure S6. Spectrum of compound **16d**, ^{1}H NMR (400 MHz, DMSO-d_6) δ 9.28 (d, *J* = 6.8 Hz, 2H), 8.84 (d, *J* = 6.8 Hz, 2H), 4.49 (s, 3H). ESI-MS analysis for [$C_{15}H_7F_{15}N_3O^+$]: Calc.: 530.0344 m/z, exp.: 530.0345 m/z, [PF_6^-]: Calc.: 144.9647 m/z, exp.: 144.9648 m/z.

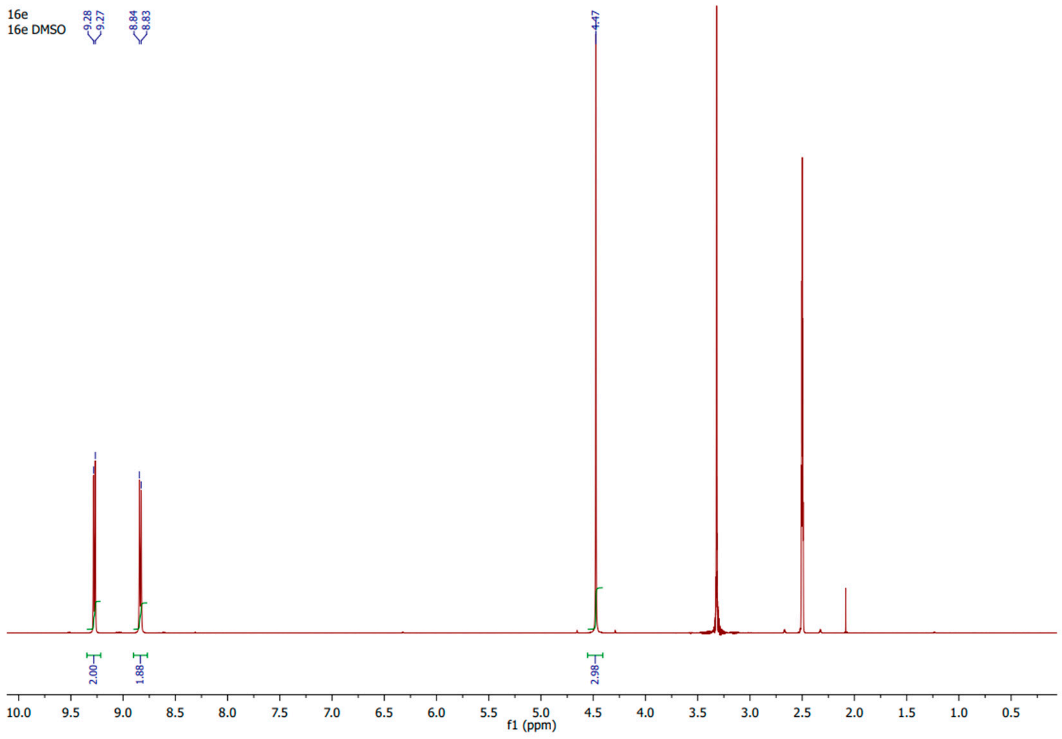

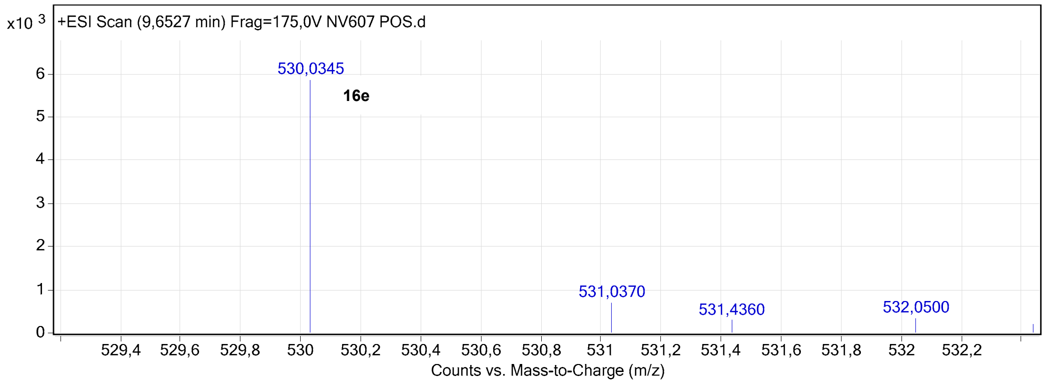

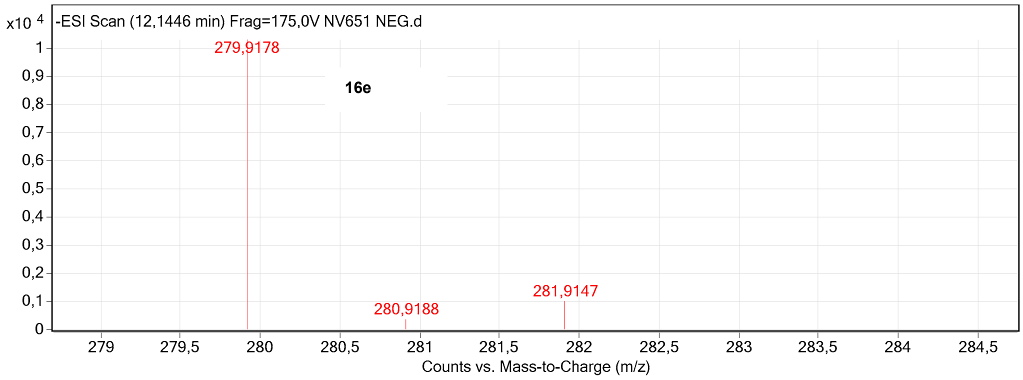

Figure S7. Spectrum of compound **16e,** ^{1}H NMR (400 MHz, DMSO-d$_6$) δ 9.27 (d, *J* = 6.8 Hz, 2H), 8.83 (d, *J* = 6.8 Hz, 2H), 4.47 (s, 3H). ESI-MS analysis for [$C_{15}H_7F_{15}N_3O^+$]: Calc.: 530.0344 m/z, exp.: 530.0345 m/z, [NTf_2^-]: Calc.: 279.9178 m/z, exp.: 279.9178 m/z.

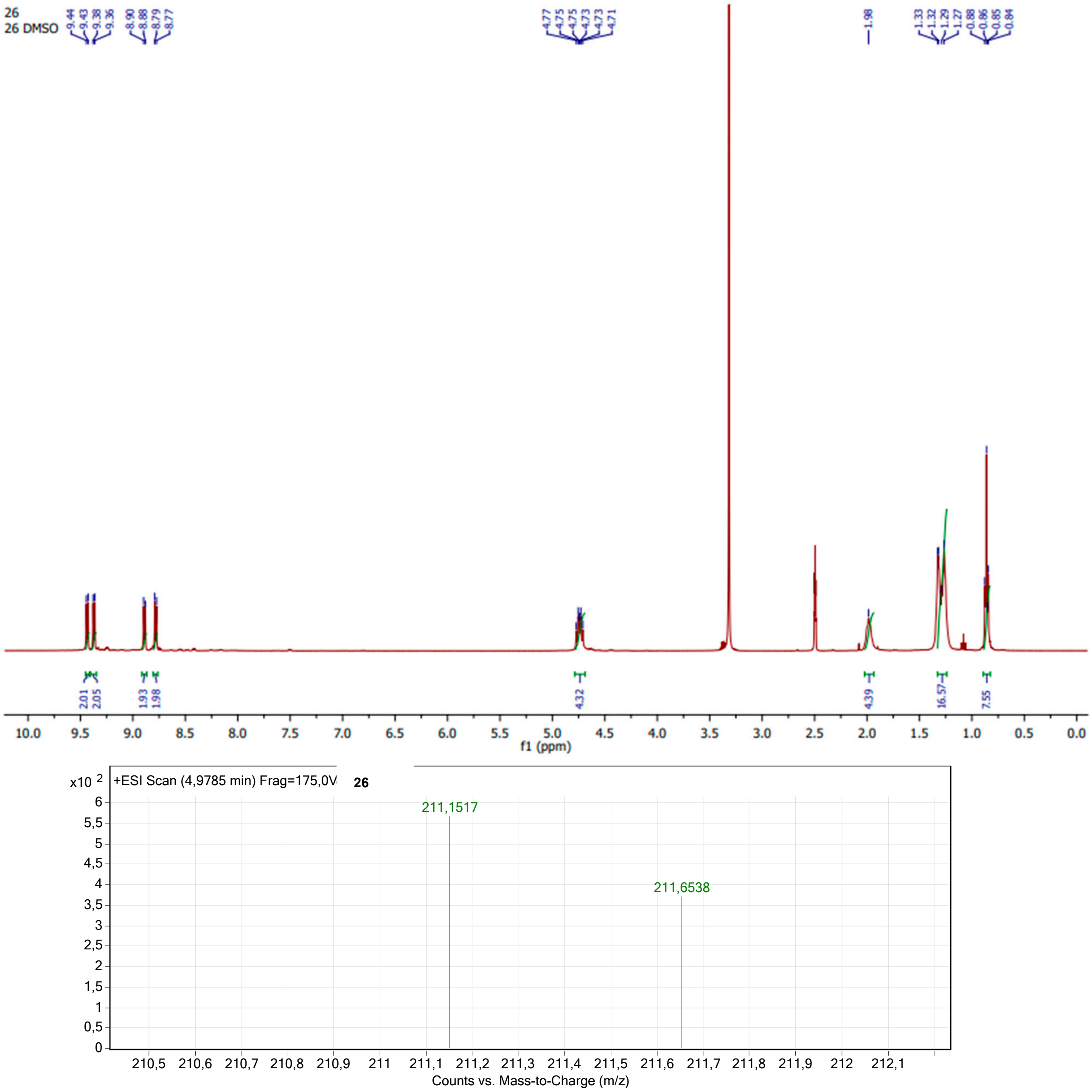

Figure S8. Spectrum of compound **26,** ^{1}H NMR (400 MHz, DMSO-d_6) δ 9.44 (d, *J* = 6.8 Hz, 2H), 9.37 (d, *J* = 6.9 Hz, 2H), 8.89 (d, *J* = 6.9 Hz, 2H), 8.78 (d, *J* = 6.8 Hz, 2H), 4.74 (m, 4H), 1.98 (s, 4 H), 1.30 (m, 16H), 0.86 (m, 6H). ESI-MS analysis for [$C_{26}H_{38}N_4O_2^{2+}$]: Calc.: 211.1517 m/z, exp.: 211.1517 m/z.

Molecule 12a

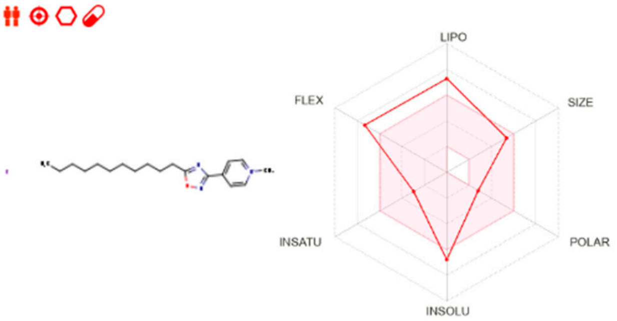

SMILES CCCCCCCCCCCc1onc(n1)c1cc[n+](cc1)C.[I-]

Physicochemical Properties	
Formula	C19H30IN3O
Molecular weight	443.37 g/mol
Num. heavy atoms	24
Num. arom. heavy atoms	11
Fraction Csp3	0.63
Num. rotatable bonds	11
Num. H-bond acceptors	3
Num. H-bond donors	0
Molar Refractivity	110.38
TPSA	42.80 Å²

Lipophilicity	
Log $P_{o/w}$ (iLOGP)	1.94
Log $P_{o/w}$ (XLOGP3)	7.25
Log $P_{o/w}$ (WLOGP)	1.64
Log $P_{o/w}$ (MLOGP)	3.49
Log $P_{o/w}$ (SILICOS-IT)	5.17
Consensus Log $P_{o/w}$	3.90

Water Solubility	
Log *S* (ESOL)	-6.77
Solubility	7.54e-05 mg/ml ; 1.70e-07 mol/l
Class	Poorly soluble
Log *S* (Ali)	-7.97
Solubility	4.71e-06 mg/ml ; 1.06e-08 mol/l
Class	Poorly soluble
Log *S* (SILICOS-IT)	-6.99
Solubility	4.52e-05 mg/ml ; 1.02e-07 mol/l
Class	Poorly soluble

Pharmacokinetics	
GI absorption	High
BBB permeant	Yes
P-gp substrate	No
CYP1A2 inhibitor	Yes
CYP2C19 inhibitor	Yes
CYP2C9 inhibitor	No
CYP2D6 inhibitor	No
CYP3A4 inhibitor	No
Log K_p (skin permeation)	-3.86 cm/s

Druglikeness	
Lipinski	Yes; 0 violation
Ghose	Yes
Veber	No; 1 violation: Rotors>10
Egan	Yes
Muegge	No; 1 violation: XLOGP3>5
Bioavailability Score	0.55

Medicinal Chemistry	
PAINS	0 alert
Brenk	2 alerts: iodine, quaternary_nitrogen_1
Leadlikeness	No; 3 violations: MW>350, Rotors>7, XLOGP3>3.5
Synthetic accessibility	3.54

Figure S9. SwissADME simulation of **12a.**

Molecule 12b

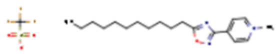

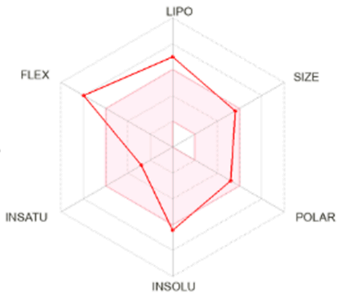

SMILES [O-]S(=O)(=O)C(F)(F)F.CCCCCCCCCCCc1onc(n1)c1cc[n+](cc1)C

Physicochemical Properties	
Formula	C20H30F3N3O4S
Molecular weight	465.53 g/mol
Num. heavy atoms	31
Num. arom. heavy atoms	11
Fraction Csp3	0.65
Num. rotatable bonds	12
Num. H-bond acceptors	9
Num. H-bond donors	0
Molar Refractivity	112.16
TPSA	108.38 Å²

Lipophilicity	
Log $P_{o/w}$ (iLOGP)	-2.92
Log $P_{o/w}$ (XLOGP3)	6.71
Log $P_{o/w}$ (WLOGP)	7.03
Log $P_{o/w}$ (MLOGP)	2.62
Log $P_{o/w}$ (SILICOS-IT)	5.17
Consensus Log $P_{o/w}$	3.72

Water Solubility	
Log *S* (ESOL)	-6.42
Solubility	1.75e-04 mg/ml ; 3.77e-07 mol/l
Class	Poorly soluble
Log *S* (Ali)	-8.79
Solubility	7.55e-07 mg/ml ; 1.62e-09 mol/l
Class	Poorly soluble
Log *S* (SILICOS-IT)	-6.99
Solubility	4.74e-05 mg/ml ; 1.02e-07 mol/l
Class	Poorly soluble

Pharmacokinetics	
GI absorption	Low
BBB permeant	No
P-gp substrate	Yes
CYP1A2 inhibitor	No
CYP2C19 inhibitor	Yes
CYP2C9 inhibitor	No
CYP2D6 inhibitor	No
CYP3A4 inhibitor	No
Log K_p (skin permeation)	-4.38 cm/s

Druglikeness	
Lipinski	Yes; 0 violation
Ghose	No; 1 violation: WLOGP>5.6
Veber	No; 1 violation: Rotors>10
Egan	No; 1 violation: WLOGP>5.88
Muegge	No; 1 violation: XLOGP3>5
Bioavailability Score	0.55

Medicinal Chemistry	
PAINS	0 alert
Brenk	3 alerts: quaternary_nitrogen_1, sulfonic_acid_2, triflate
Leadlikeness	No; 3 violations: MW>350, Rotors>7, XLOGP3>3.5
Synthetic accessibility	3.81

Figure S10. SwissADME simulation of **12b.**

Molecule 17a

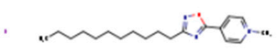

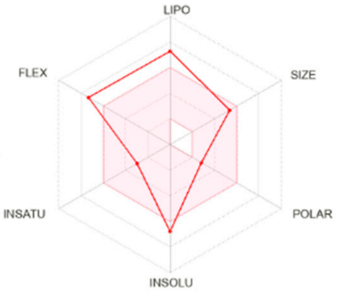

SMILES CCCCCCCCCCCc1noc(n1)c1cc[n+](cc1)C.[I-]

Physicochemical Properties	
Formula	C19H30IN3O
Molecular weight	443.37 g/mol
Num. heavy atoms	24
Num. arom. heavy atoms	11
Fraction Csp3	0.63
Num. rotatable bonds	11
Num. H-bond acceptors	3
Num. H-bond donors	0
Molar Refractivity	110.38
TPSA	42.80 Å²

Lipophilicity	
Log $P_{o/w}$ (iLOGP)	1.65
Log $P_{o/w}$ (XLOGP3)	7.25
Log $P_{o/w}$ (WLOGP)	1.64
Log $P_{o/w}$ (MLOGP)	3.89
Log $P_{o/w}$ (SILICOS-IT)	5.17
Consensus Log $P_{o/w}$	3.92

Water Solubility	
Log *S* (ESOL)	-6.77
Solubility	7.54e-05 mg/ml ; 1.70e-07 mol/l
Class	Poorly soluble
Log *S* (Ali)	-7.97
Solubility	4.71e-06 mg/ml ; 1.06e-08 mol/l
Class	Poorly soluble
Log *S* (SILICOS-IT)	-6.99
Solubility	4.52e-05 mg/ml ; 1.02e-07 mol/l
Class	Poorly soluble

Pharmacokinetics	
GI absorption	High
BBB permeant	Yes
P-gp substrate	No
CYP1A2 inhibitor	Yes
CYP2C19 inhibitor	Yes
CYP2C9 inhibitor	No
CYP2D6 inhibitor	No
CYP3A4 inhibitor	No
Log K_p (skin permeation)	-3.86 cm/s

Druglikeness	
Lipinski	Yes; 0 violation
Ghose	Yes
Veber	No; 1 violation: Rotors>10
Egan	Yes
Muegge	No; 1 violation: XLOGP3>5
Bioavailability Score	0.55

Medicinal Chemistry	
PAINS	0 alert
Brenk	2 alerts: iodine, quaternary_nitrogen_1
Leadlikeness	No; 3 violations: MW>350, Rotors>7, XLOGP3>3.5
Synthetic accessibility	3.71

Figure S11. SwissADME simulation of **17a.**

Molecule 17b

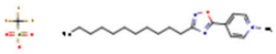

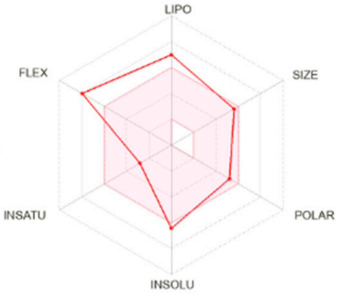

SMILES [O-]S(=O)(=O)C(F)(F)F.CCCCCCCCCCCc1noc(n1)c1cc[n+](cc1)C

Physicochemical Properties	
Formula	C20H30F3N3O4S
Molecular weight	465.53 g/mol
Num. heavy atoms	31
Num. arom. heavy atoms	11
Fraction Csp3	0.65
Num. rotatable bonds	12
Num. H-bond acceptors	9
Num. H-bond donors	0
Molar Refractivity	112.16
TPSA	108.38 Å²

Lipophilicity	
Log $P_{o/w}$ (iLOGP)	-3.14
Log $P_{o/w}$ (XLOGP3)	6.71
Log $P_{o/w}$ (WLOGP)	7.03
Log $P_{o/w}$ (MLOGP)	3.02
Log $P_{o/w}$ (SILICOS-IT)	5.17
Consensus Log $P_{o/w}$	3.76

Water Solubility	
Log *S* (ESOL)	-6.42
Solubility	1.75e-04 mg/ml ; 3.77e-07 mol/l
Class	Poorly soluble
Log *S* (Ali)	-8.79
Solubility	7.55e-07 mg/ml ; 1.62e-09 mol/l
Class	Poorly soluble
Log *S* (SILICOS-IT)	-6.99
Solubility	4.74e-05 mg/ml ; 1.02e-07 mol/l
Class	Poorly soluble

Pharmacokinetics	
GI absorption	Low
BBB permeant	No
P-gp substrate	Yes
CYP1A2 inhibitor	No
CYP2C19 inhibitor	Yes
CYP2C9 inhibitor	No
CYP2D6 inhibitor	No
CYP3A4 inhibitor	No
Log K_p (skin permeation)	-4.38 cm/s

Druglikeness	
Lipinski	Yes; 0 violation
Ghose	No; 1 violation: WLOGP>5.6
Veber	No; 1 violation: Rotors>10
Egan	No; 1 violation: WLOGP>5.88
Muegge	No; 1 violation: XLOGP3>5
Bioavailability Score	0.55

Medicinal Chemistry	
PAINS	0 alert
Brenk	3 alerts: quaternary_nitrogen_1, sulfonic_acid_2, triflate
Leadlikeness	No; 3 violations: MW>350, Rotors>7, XLOGP3>3.5
Synthetic accessibility	3.97

Figure S12. SwissADME simulation of **17b.**

Molecule 27

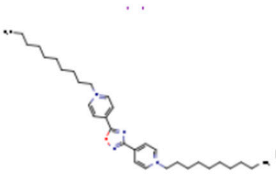

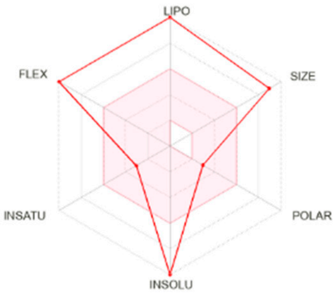

SMILES CCCCCCCCCC[n+]1ccc(cc1)c1onc(n1)c1cc[n+](cc1)CCCCCCCCCC.[I-].[I-]

Physicochemical Properties	
Formula	C32H50I2N4O
Molecular weight	760.57 g/mol
Num. heavy atoms	39
Num. arom. heavy atoms	17
Fraction Csp3	0.62
Num. rotatable bonds	20
Num. H-bond acceptors	3
Num. H-bond donors	0
Molar Refractivity	186.92
TPSA	46.68 Å²

Lipophilicity	
Log $P_{o/w}$ (iLOGP)	-0.80
Log $P_{o/w}$ (XLOGP3)	12.17
Log $P_{o/w}$ (WLOGP)	2.27
Log $P_{o/w}$ (MLOGP)	5.64
Log $P_{o/w}$ (SILICOS-IT)	8.76
Consensus Log $P_{o/w}$	5.61

Water Solubility	
Log *S* (ESOL)	-11.23
Solubility	4.53e-09 mg/ml ; 5.95e-12 mol/l
Class	Insoluble
Log *S* (Ali)	-13.16
Solubility	5.26e-11 mg/ml ; 6.91e-14 mol/l
Class	Insoluble
Log *S* (SILICOS-IT)	-11.39
Solubility	3.13e-09 mg/ml ; 4.11e-12 mol/l
Class	Insoluble

Pharmacokinetics	
GI absorption	High
BBB permeant	Yes
P-gp substrate	Yes
CYP1A2 inhibitor	No
CYP2C19 inhibitor	No
CYP2C9 inhibitor	No
CYP2D6 inhibitor	No
CYP3A4 inhibitor	No
Log K_p (skin permeation)	-2.30 cm/s

Druglikeness	
Lipinski	No; 2 violations: MW>500, MLOGP>4.15
Ghose	No; 3 violations: MW>480, MR>130, #atoms>70
Veber	No; 1 violation: Rotors>10
Egan	Yes
Muegge	No; 3 violations: MW>600, XLOGP3>5, Rotors>15
Bioavailability Score	0.17

Medicinal Chemistry	
PAINS	0 alert
Brenk	2 alerts: iodine, quaternary_nitrogen_1
Leadlikeness	No; 3 violations: MW>350, Rotors>7, XLOGP3>3.5
Synthetic accessibility	4.97

Figure S13. SwissADME simulation of **27.**

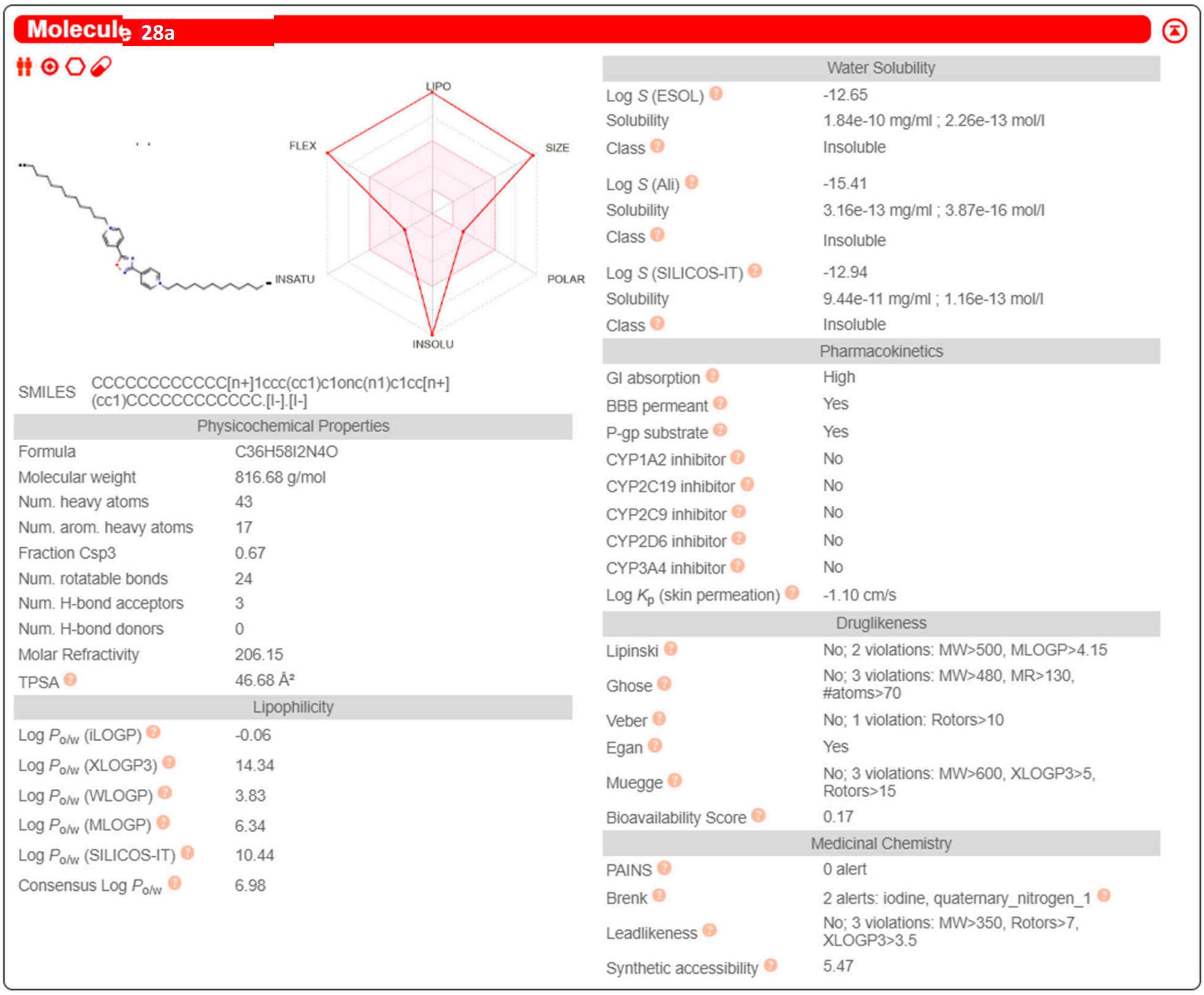

Figure S14. SwissADME simulation of **28a.**

Structural characterization analysis of previously reported compounds

Synthesis and characterization are in agreement with the previously reported data [1-4]

***N*-Methyl-4-(5-pentadecafluoroheptyl-1,2,4-oxadiazol-3-yl)pyridinium iodide (11a)** Yield= 62%, yellow solid, m.p. 99-100 °C, ^{1}H NMR (300 MHz, CD_3CN) δ (ppm)= 9.03 (d, *J* = 6.5 Hz, 2H), 8.60 (d, *J* = 6.5 Hz, 2H), 4.41 (s, 3H).

***N*-Methyl-4-(5-pentadecafluoroheptyl-1,2,4-oxadiazol-3-yl)pyridinium trifluoromethanesulfonate (11b)** Yield= 83%, red solid, m.p. 130-132 °C, ^{1}H NMR (CD_3CN) δ (ppm)= 8.86 (d, *J* = 6.5 Hz, 2H), 8.59 (d, *J* = 6.5 Hz, 2H), 4.39 (s, 3H).

***N*-Methyl-4-(5-undecyl-1,2,4-oxadiazol-3-yl)pyridinium Iodide (12a)** Yield=99%, yellow oil, ^{1}H NMR (300 MHz, CD_3CN) δ (ppm)= 8.98 (d, *J*=6.3 Hz, 2H), 8.61 (d, *J*=6.3 Hz, 2H), 4.49 (s, 3H), 3.10 (t, *J*=7.6 Hz, 2H), 1.92 (m, 2H), 1.39 (m, 16H), 0.93 (t, J= 6.6 Hz, 3H).

***N*-Methyl-4-(5-undecyl-1,2,4-oxadiazole-3-yl)pyridinium trifluoromethanesulfonate (12b)** Yield=71%, yellow solid, m.p. 41-42 °C, ^{1}H NMR (300 MHz, CD_3CN) δ (ppm)= 8.80 (d, *J*= 6.6 Hz, 2H), 8.51 (d, *J*= 6.6 Hz, 2H), 4.36 (s, 3H), 3.02 (t, *J*= 7.5 Hz, 2H), 1.84 (m, 2H), 1.31 (m, 16H), 0.86 (t, *J*= 6.2 Hz, 3H).

***N*-Methyl-3-(5-pentadecafluoroheptyl-1,2,4-oxadiazol-3-yl)pyridinium iodide (14a):** Yield= 71%, red solid m.p. 123-125 °C, ^{1}H NMR (300 MHz, CD_3CN) δ (ppm)= 9.37 (s, 1H), 9.06 (d, *J* = 8.0 Hz, 1H), 8.89 (d, *J* = 6.0 Hz, 1H), 8.23 (dd, *J* = 6.0 and 8.0 Hz, 1H), 4.43 (s, 3H).

***N*-Methyl-3-(3-pentadecafluoroheptyl-1,2,4-oxadiazol-5-yl)pyridinium iodide (15a)** Yield= 63%, m.p. 150-152 °C, brown solid, ^{1}H NMR (300 MHz, CD_3CN) δ (ppm)= 9.51 (s, 1H), 9.11 (d, *J* = 8.0 Hz, 1H), 9.02 (d, *J* = 6.0 Hz, 1H), 8.30 (dd, *J* = 6.0 and 8.0 Hz, 1H), 4.47 (s, 3H).

***N*-Methyl-3-(3-pentadecafluoroheptyl-1,2,4-oxadiazol-5-yl)pyridinium trifluoromethanesulfonate (15b)** Yield= 62%, m.p. 110-112 °C, yellow solid, ^{1}H NMR (300 MHz, CD_3CN) δ (ppm)= 9.46 (s, 1H), 9.11 (d, *J* = 8.0 Hz, 1H), 8.91 (d, *J* = 6.0 Hz, 1H), 8.27 (dd, *J* = 6.0 and 8.0 Hz, 1H), 4.44 (s, 3H).

***N*-Methyl-4-(3-pentadecafluoroheptyl-1,2,4-oxadiazol-5-yl)pyridinium iodide (16a)** Yield= 90%, m.p. 95-97 °C, red solid, ^{1}H NMR (300 MHz, CD_3CN) δ (ppm)= 9.03 (d, *J* = 6.0 Hz, 2H), 8.65 (d, *J* = 6.0 Hz, 2H), 4.46 (s, 3H).

***N*-Methyl-4-(3-pentadecafluoroheptyl-1,2,4-oxadiazol-5-yl)pyridinium trifluoromethanesulfonate (16b)** Yield= 91%, m.p. 99-102 °C, brown solid, ^{1}H NMR (300 MHz, CD_3CN) δ (ppm)= 8.93 (d, *J* = 6.5 Hz, 2H), 8.63 (d, *J* = 6.5 Hz, 2H), 4.42 (s, 3H).

***N*-Methyl-4-(3-undecyl-1,2,4-oxadiazol-5-yl)pyridinium iodide (17a)** Yield=58%, m.p= 94-96 °C, yellow solid, ^{1}H NMR (300 MHz, CD_3CN) δ (ppm)= 8.9 (d, *J*= 6.6 Hz, 2H), 8.55 (d, *J*= 6.6 Hz, 2H), 4.40 (s, 3H), 2.85 (t, *J*= 7.4 Hz, 2H), 1.77 (m, 2H), 1.26 (m, 16H), 0.87 (t, *J*= 6.8 Hz, 3H).

***N*-Methyl-4-(3-undecyl-1,2,4-oxadiazol-5-yl)pyridinium trifluoromethanesulfonate (17b)** Yield=40%, m.p. 105 °C, red solid, ^{1}H NMR (300 MHz, CD_3CN) δ (ppm)= 8.85 (d, *J*= 6.5 Hz, 2H), 8.54 (d, *J*= 6.5 Hz, 2), 4.37 (s, 3H), 2.85 (t, *J*= 7.3 Hz, 2H), 1.78 (m, 2H), 1.27 (m, 16H), 0.87 (t, *J*= 6.1 Hz, 3H).

***N*-Methyl-4-(3-heptyl-1,2,4-oxadiazol-5-yl)pyridinium iodide (18a)** Yield=73%, yellow solid, m.p. 84-85 °C, ^{1}H NMR (300 MHz, CD_3CN) δ (ppm)= 8.98 (d, *J*= 6.2 Hz, 2H), 8.57 (d, *J*= 6.2 Hz, 2H), 4.43 (s, 3H), 2.85 (t, *J*= 7.4 Hz, 2H), 1.78 (m, 2H), 1.32 (m, 8H), 0.87 (t, *J*= 6.3 Hz, 3H).

***N*-Methyl-4-(3-heptyl-1,2,4-oxadiazol-5-yl)pyridinium trifluoromethanesulfonate (18b)** Yield= 84%, red solid, m.p. 96-97 °C, ^{1}H NMR (300 MHz, CD_3CN) δ (ppm)= 8.89 (d, *J*= 6.4 Hz, 2H), 8.55 (d, *J*= 6.4 Hz, 2H), 4.40 (s, 3H), 2.85 (t, *J*= 7.4 Hz, 2H), 1.78 (m, 2H), 1.32 (m, 8H), 0.87 (t, *J*= 6.4 Hz, 3H).

***N*-Methyl-4-(3-pentadecafluoroheptyl-1-methyl-1,2,4-triazol-5-yl)pyridinium iodide (19a)** Yield= 96% m.p. 165-167 °C, red solid, ^{1}H NMR (300 MHz, CD_3CN) δ (ppm)= 8.87 (d, *J* = 6.0 Hz, 2H), 8.38 (d, *J* = 6.0 Hz, 2H), 4.37 (s, 3H), 4.16 (s, 3H).

***N*-Methyl-4-(3-pentadecafluoroheptyl-1-methyl-1,2,4-triazol-3-yl)pyridinium trifluoromethanesulfonate (19b)** Yield= 46%, yellow solid, m.p. 126-129 °C, ^{1}H NMR (300 MHz, CD_3CN) δ (ppm)= 8.83 (d, *J* = 6.5 Hz, 2H), 8.38 (d, *J* = 6.5 Hz, 2H), 4.39 (s, 3H), 4.18 (s, 3H).

***N*-Methyl-4-(5-pentadecafluoroheptyl-1-methyl-1,2,4-triazol-3-yl)pyridinium iodide (20a)** Yield= 99%, red solid, m.p. 188-190 °C, ^{1}H NMR (300 MHz, $CDCl_3$) δ (ppm)= 9.42 (d, *J* = 6.5 Hz, 2H), 8.62 (d, *J* = 6.5 Hz, 2H), 4.77 (s, 3H), 4.23 (s, 3H).

***N*-Methyl-4-(5-pentadecafluoroheptyl-1-methyl-1,2,4-triazol-3-yl)pyridinium trifluoromethanesulfonate (20b)** Yield= 49%, brown solid, m.p. 113-116 °C, ^{1}H NMR (300 MHz, CD_3CN) δ(ppm)= 8.72 (d, *J* = 6.5 Hz, 2H), 8.51 (d, *J* = 6.5 Hz, 2H), 4.34 (s, 3H), 4.19 (s, 3H).

1-(3,3,4,4,5,5,6,6,7,7,8,8,9,9,10,10,10-heptadecafluorodecyl)-4-(3-(pyridin-4-yl)-1,2,4-oxadiazol-5-yl)pyridin-1-ium (22)

Yield= 40%, red solid, m.p. 221°C, ^{1}H NMR (400 MHz, DMSO-d_6) δ(ppm)= 9.45 (d, *J* = 7.0 Hz, 2H), 8.97 (dd, *J* = 4.4, 1.7 Hz, 2H), 8.83 (d, *J* = 7.0 Hz, 2H), 8.15 (dd, *J* = 4.4, 1.7 Hz, 2H), 5.25 – 4.98 (m, 4H).

4,4'-(1,2,4-oxadiazole-3,5-diyl)bis(pyridin-1-ium) 2,2,3,3,4,4,5,5,6,6,7,7-dodecafluorooctanedioate (23a)

Yield= 67%, white solid, m.p. 185 °C, ^{1}H NMR (400 MHz, DMSO-d_6) δ 8.95 (dd, *J* = 4.6, 1.6 Hz, 2H), 8.91 (dd, *J* = 4.6, 1.6 Hz, 2H), 8.14 (m, 4H).

4,4'-(1,2,4-oxadiazole-3,5-diyl)bis(pyridin-1-ium) 2,2,3,3,4,4,5,5,6,6,7,7,8,8,9,9-hexadecafluorodecanedioate (23b)

Yield= 58%, white solid, m.p. 201 °C, ^{1}H NMR (400 MHz, DMSO-d_6) δ 8.95 (dd, *J* = 4.4, 1.7 Hz, 2H), 8.90 (dd, *J* = 4.5, 1.7 Hz, 2H), 8.15 (dd, *J* = 4.4, 1.7 Hz, 2H), 8.12 (dd, *J* = 4.5, 1.7 Hz, 2H).

4,4'-(1,2,4-oxadiazole-3,5-diyl)bis(pyridin-1-ium) 2,2,3,3,4,4,5,5,6,6,7,7,7-tridecafluoroheptanoate (23c)

Yield=45% , white solid, m.p. 130°C, ^{1}H NMR (400 MHz, DMSO-d_6) δ 8.95 (d, *J* = 5.0 Hz, 2H), 8.91 (d, *J* =5.0 Hz, 2H), 8.15 (d, *J* = 5.8 Hz, 2H), 8.13 (d, *J* = 5.8 Hz, 2H).

4,4'-(1,2,4-oxadiazole-3,5-diyl)bis(1-methylpyridin-1-ium) iodide (24)

Yield= 76%, brown solid, m.p. 143 °C , ^{1}H NMR (400 MHz, DMSO-d_6) δ 9.29 (d, *J* = 6.8 Hz, 2H), 9.23 (d, *J* = 6.8 Hz, 2H), 8.83 (d, *J* = 6.8 Hz, 2H), 8.73 (d, *J* = 6.8 Hz, 2H), 4.47 (s, 3H), 4.45 (s, 3H).

4,4'-(1,2,4-oxadiazole-3,5-diyl)bis(1-butylpyridin-1-ium) iodide (25)
Yield= 80%, red solid, m.p. 166°C, ^{1}H NMR (400 MHz, DMSO-d_6) δ 9.43 (d, *J* = 6.8 Hz, 2H), 9.36 (d, *J* = 6.8 Hz, 2H), 8.88 (d, *J* = 6.8 Hz, 2H), 8.78 (d, *J* = 6.8 Hz, 2H), 4.75 (dt, *J* = 10.0, 7.4 Hz, 4H), 2.01 – 1.91 (m, 4H), 1.41 – 1.28 (m, 4H), 0.94 (t, *J* = 7.4 Hz, 6H).

4,4'-(1,2,4-oxadiazole-3,5-diyl)bis(1-decylpyridin-1-ium) iodide (27): Yield = 52.9%, red solid, m.p. 170 °C, ^{1}H NMR (300 MHz, DMSO-d_6) δ(ppm)= 9.44 (d, *J* = 6.9 Hz, 2H), 9.38 (d, *J* = 6.9 Hz, 2H), 8.90 (d, *J* = 6.6 Hz, 2H), 8.79 (d, *J* = 6.6 Hz, 2H), 4.75 (t, *J* = 9 Hz, 2H), 4.73 (t, *J* = 9 Hz, 2H), 2.08 – 1.90 (m, 4H), 1.34 – 1.25 (m, 28H), 0.85 (t, *J* = 6.5 Hz, 6H).

4,4'-(1,2,4-oxadiazole-3,5-diyl)bis(1-dodecylpyridin-1-ium) iodide (28a) Yield = 50.6%, red solid, m.p. 95 °C, ^{1}H NMR (300 MHz DMSO-d_6,) δ (ppm)= 9.45 (d, *J* = 6.9 Hz, 2H), 9.38 (d, *J* = 6.9 Hz, 2H), 8.90 (d, *J* = 6.6 Hz, 2H), 8.79 (d, *J* = 6.6 Hz, 2H), 4.76 (t, *J* = 9 Hz, 2H), 4.74 (t, *J* = 9 Hz, 2H), 2.06 – 1.91 (m, 4H), 1.34 – 1.26 (m, 36H), 0.85 (t, *J* = 6.5 Hz, 6H).

4,4'-(1,2,4-oxadiazole-3,5-diyl)bis(1-dodecylpyridin-1-ium) bis((trifluoromethyl)sulfonyl)amide (28b) Yield = 51.1%, white solid, m.p. 130° C, ^{1}H NMR (300 MHz, DMSO-d_6) δ(ppm)= 9.42 (d, *J* = 6.9 Hz, 2H), 9.38 (d, *J* = 6.9 Hz, 2H), 8.90 (d, *J* = 6.6 Hz, 2H), 8.79 (d, *J* = 6.6 Hz, 2H), 4.74 (t, *J* = 9 Hz, 2H), 4.72 (t, *J* = 9 Hz, 2H), 2.06 – 1.91 (m, 4H), 1.34 – 1.26 (m, 36H), 0.85 (t, *J* = 6.5 Hz, 6H).

4,4'-(1,2,4-oxadiazole-3,5-diyl)bis(1-tetradecylpyridin-1-ium) bromide (29a) Yield = 75.2%, yellow solid, m.p. 190 °C, ^{1}H NMR (300 MHz, DMSO-d_6) δ(ppm)= 9.44 (d, *J* = 6.9 Hz, 2H), 9.38 (d, *J* = 6.9 Hz, 2H), 8.89 (d, *J* = 6.6 Hz, 2H), 8.78 (d, *J* = 6.6 Hz, 2H), 4.75 (t, *J* = 9 Hz, 2H), 4.73 (t, *J* = 9 Hz, 2H), 2.03 – 1.89 (m, 4H), 1.32 – 1.23 (m, 44H), 0.84 (t, *J* = 6.5 Hz, 6H).

4,4'-(1,2,4-oxadiazole-3,5-diyl)bis(1-tetradecylpyridin-1-ium) bis((trifluoromethyl)sulfonyl)amide (29b) Yield = 46.5%, white solid, m.p. 210°C, ^{1}H NMR (300 MHz, DMSO-d_6) δ(ppm)= 9.42 (d, *J* = 6.9 Hz, 2H), 9.38 (d, *J* = 6.9 Hz, 2H), 8.90 (d, *J* = 6.6 Hz, 2H), 8.79 (d, *J* = 6.6 Hz, 2H), 4.74 (t, *J* = 9 Hz, 2H), 4.72 (t, *J* = 9 Hz, 2H), 2.06 – 1.91 (m, 4H), 1.34 – 1.26 (m, 44H), 0.85 (t, J = 6.5 Hz, 6H).

4,4'-(1,2,4-oxadiazole-3,5-diyl)bis(1-(3,3,4,4,5,5,6,6,7,7,8,8,9,9,10,10,10-heptadecafluorodecyl) pyridin-1-ium) iodide (30a) Yield = 75.8%, yellow solid, m.p. 223 °C, ^{1}H NMR (300 MHz, DMSO-d_6) δ(ppm)= 9.55 (d, *J* = 6.9 Hz, 2H), 9.49 (d, *J* = 6.9 Hz, 2H), 8.96 (d, *J* = 6.6 Hz, 2H), 8.85 (d, *J* = 6.6 Hz, 2H), 5.17 (t, *J* = 9 Hz, 2H), 5.14 (t, *J* = 9 Hz, 2H), 2.90 – 2.71 (m, 4H).

4,4'-(1,2,4-oxadiazole-3,5-diyl)bis(1-(3,3,4,4,5,5,6,6,7,7,8,8,9,9,10,10,10-heptadecafluorodecyl) pyridin-1-ium) iodide (30b) Yield = 75.8%, yellow solid, m.p. 235 °C, ^{1}H NMR (300 MHz, DMSO-d_6) δ(ppm)= 9.55 (d, *J* = 6.9 Hz, 2H), 9.49 (d, *J* = 6.9 Hz, 2H), 8.96 (d, *J* = 6.6 Hz, 2H), 8.85 (d, *J* = 6.6 Hz, 2H), 5.17 (t, *J* = 9 Hz, 2H), 5.14 (t, *J* = 9 Hz, 2H), 2.90 – 2.71 (m, 4H).
